# What Controls the Water Vapor Isotopic Composition Near the Surface of Tropical Oceans? Results From an Analytical Model Constrained by Large‐Eddy Simulations

**DOI:** 10.1029/2020MS002106

**Published:** 2020-08-12

**Authors:** Camille Risi, Caroline Muller, Peter Blossey

**Affiliations:** ^1^ Laboratoire de Meteorologie Dynamique, IPSL, CNRS, Ecole Normale Superieure Sorbonne Universite, PSL Research University Paris France; ^2^ Department of Atmospheric Sciences University of Washington Seattle WA USA

**Keywords:** water isotopes, convection, large‐eddy simulation

## Abstract

The goal of this study is to understand the mechanisms controlling the isotopic composition of the water vapor near the surface of tropical oceans, at the scale of about a hundred kilometers and a month. In the tropics, it has long been observed that the isotopic compositions of rain and vapor near the surface are more depleted when the precipitation rate is high. This is called the “amount effect.” Previous studies, based on observations or models with parameterized convection, have highlighted the roles of deep convective and mesoscale downdrafts and rain evaporation. But the relative importance of these processes has never been quantified. We hypothesize that it can be quantified using an analytical model constrained by large‐eddy simulations. Results from large‐eddy simulations confirm that the classical amount effect can be simulated only if precipitation rate changes result from changes in the large‐scale circulation. We find that the main process depleting the water vapor compared to the equilibrium with the ocean is the fact that updrafts stem from areas where the water vapor is more enriched. The main process responsible for the amount effect is the fact that when the large‐scale ascent increases, isotopic vertical gradients are steeper, so that updrafts and downdrafts deplete the subcloud layer more efficiently.

## Key Points

- The precipitation is more depleted when the precipitation rate is higher only if it is associated with stronger large‐scale ascent
- Updrafts are responsible for most of the depletion of the near‐surface water vapor relative to equilibrium with the ocean
- With large‐scale ascent, the near‐surface water vapor is more depleted because updrafts export enriched water more efficiently

## 1 Introduction

The isotopic composition of water is the relative proportion of heavy (*HDO*, H ${}_{2}^{18}$ O) and light (H ${}_{2}^{16}$ O) water molecules. The enrichment of heavy isotopes (e.g., *HDO*) is commonly expressed as $\delta D=\left(R/R_{SMOW}-1\right)\times 1,000$ in ‰, where *R* is the ratio of deuterium over hydrogen atoms in the water, and SMOW is the Standard Mean Ocean Water reference. Recorded in precipitation archives such as ice or speleothems, the isotopic composition of water provides information on past climatic variations (Jouzel et al., 2003; Thompson et al., 2000; Wang et al., 2008). Measured in water vapor, it shows variations that might be useful to better understand atmospheric and hydrological processes (Galewsky & Samuels‐Crow, 2014; Galewsky, Steen‐Larsen, et al. 2016; Worden et al., 2007) or evaluate general circulation models (GCMs) (Bony et al., 2008; Field et al., 2014). For these applications, it is necessary to understand the mechanisms controlling the isotopic composition of water.

A first step is to understand the mechanisms controlling the isotopic composition of water vapor near the surface of tropical (30°S to 30°N) oceans, since it then supplies water to all other regions of the atmosphere (Risi et al., 2019). Indeed, this water vapor is an important moistening source to air masses traveling to land regions (Gimeno et al., 2010; Ent & Savenije, 2013) and toward higher latitudes (Ciais et al., 1995; Delaygue et al., 2000). It is also ultimately the only source of water vapor in the tropical free troposphere, since water vapor in the free troposphere ultimately originates from convective detrainment (Sherwood, 1996), and convection ultimately feeds from the air close to the surface (Bony et al., 2008). In addition, the precipitation isotopic composition often varies in concert with the water vapor (Aemisegger et al., 2012; Graf et al., 2019; Nlend et al., 2020; Shi et al., 2020; Tremoy et al., 2012). Therefore, the water vapor isotopic composition near the surface of tropical oceans serves as an initial condition for the isotopic composition in land waters and in the tropospheric water vapor everywhere on Earth. In this study, we thus focus on the subcloud layer (SCL), that is, the first few hundreds of meters above the ocean surface and below the clouds. Also, we focus on the isotopic composition of the SCL at spatial scales of about a hundred kilometers, and at time scales of about a month.

In the tropics at such time scales, it has long been observed that the isotopic composition of rain and vapor near the surface is more depleted in heavy isotopes when the precipitation rate is high. This is called the “amount effect” (Dansgaard, 1964; Rozanski et al., 1993). Previous studies have aimed at understanding the mechanisms of this effect, from observations (Kurita, 2013; Worden et al., 2007), simple distillation or mixing line models (Worden et al., 2007), simple box models of convective systems (Kurita, 2013; Tremoy et al., 2014), general circulation models (GCMs) with parameterized convection (Lee et al., 2007) or single column versions of these GCMs (Risi et al., 2008). These studies highlighted the role of deep convective or mesoscale downdrafts (Kurita, 2013; Kurita et al., 2011; Risi et al., 2008; Risi, Bony, Vimeux, Chong, et al. 2010) and of rain evaporation (Field et al., 2010; Worden et al., 2007). But the relative importance of these processes has never been quantified. The goal of this study is thus to quantify the relative importance of these processes.

In the meanwhile, several studies highlighted the importance of weak downdrafts in the environment (i.e., outside convective systems) and of updrafts in determining the moist static energy budget of the SCL (Thayer‐Calder & Randall, 2015; Torri & Kuang, 2016a). Therefore, all kinds of drafts may potentially contribute to the amount effect. To simulate explicitly all these drafts, here we use large‐eddy simulations (LES) (Randall, Krueger, et al. 2003). By explicitly resolving convective motions, these LES avoid the numerous simplifications or assumptions that are needed in convective parameterization (Del Genio, 2012; Rio et al., 2019) and that are responsible for a significant part of biases in the present climate simulated by GCMs and of intermodel spread in climate change projections (Randall, Khairoutdinov, et al. 2003; Stevens & Bony, 2013; Webb et al., 2015).

Several high‐resolution isotope‐enabled models have appeared in recent years, for example, Wei et al. (2018). An isotope‐enabled version of SAM (Blossey et al., 2010) was used to study the amount effect (Moore et al., 2014). Based on a column‐integrated water budget, they concluded that the amount effect is consistent with a larger proportion of precipitation being derived from depleted free tropospheric moisture than from enriched water evaporated from the ocean surface, as convection becomes more intense. This column‐integrated water budget view, however, does not tell by what mechanisms the SCL water vapor becomes more depleted.

Therefore, the goal of this article is to understand the mechanisms that deplete the SCL, in average over an LES domain (about a hundred kilometers). Specifically, what processes deplete the SCL water vapor compared to what we would expect if the water vapor was in equilibrium with the ocean? What processes deplete the SCL more and more as convection becomes more intense? We hypothesize that these questions can be quantitatively addressed using an analytical model of the SCL water budget that is constrained by LES simulations. Section 2 describe the LES simulations and their results, and then section 3 describe the analytical model and its results. Conclusions are offered in section 4.

## 2 LES Simulations

### 2.1 LES Model

We use the System for Atmospheric Modeling (SAM) nonhydrostatic model (Khairoutdinov & Randall, 2003), version 6.10.9, which is enabled with water isotopes (Blossey et al., 2010). This model solves anelastic conservation equations for momentum, mass, energy and water, which is present in the model under six phases: water vapor, cloud liquid, cloud ice, precipitating liquid, precipitating snow, and precipitating graupel. The subgrid‐scale fluxes are parameterized based on Smagorinsky's eddy diffusivity model. The Rapid Radiative Transfer Model for Global climate model applications (RRTMG) radiation scheme is used (Blossey et al., 2013). Advection is represented by the fifth‐order scheme of (Yamaguchi et al., 2011). We use the bulk, mixed‐phase microphysical parameterization from Thompson et al. (2008) in which water isotopes were implemented (Moore et al., 2016).

### 2.2 Simulations

All the simulations are listed in Table 1. The control simulation (“ctrl”) is three‐dimensional, with a doubly periodic domain of 96 km  × 96 km. The horizontal resolution is 750 m. There are 96 vertical levels. The simulation is run in radiative‐convective equilibrium over an ocean surface. The sea surface temperature (SST) is 30°C. There is no rotation and no diurnal cycle; the latter is removed by using an insolation that is constant in space and time, with exactly the same incident flux and zenith angle as in Tompkins and Craig (1998) (halved solar constant 685 W · m^−2^ and zenith angle set to 51.7°). In this simulation, there is no large‐scale circulation.

**Table 1:** Configuration of the SAM Simulations Analyzed in This Study: Sea Surface Temperature (SST), Maximum Large‐Scale Vertical Velocity (*ω*_*LS*_(*p*_*max*_)), Altitude of Maximum Large‐Scale Vertical Velocity (*p*_*max*_), Horizontal Resolution (Δ*x*), and Horizontal Domain Size

Name	SST(°C)	*ω*_*LS*_(*p*_*max*_)(hPa/day)	*p*_*max*_(hPa)	Δ*x* (m)	domain size(km)	*z*_*T*_ (m)
ctrl	30	0	none	750	96	411.6
*ω*_*LS*_−60	30	−60	500	750	96	204.8
*ω*_*LS*_−20	30	−20	500	750	96	302.8
*ω*_*LS*_+20	30	+20	500	750	96	665.8
26C	26	0	none	750	96	532.3
33C	33	0	none	750	96	411.6
*p*400	30	‐60	400	750	96	302.8
*p*600	30	‐60	600	750	96	204.8
200m	30	0	none	200	25.6	411.6

*Note*. The last column indicates the simulated altitude *z*_*T*_ of the SCL top.

To compare simulations with different convective intensities, we prescribe a large‐scale vertical velocity profile, *ω*_*LS*_, which is used to compute large‐scale tendencies in temperature, humidity and water vapor isotopic composition. We compute large‐scale vertical advection by a simple upstream scheme (Godunov, 1959). In the computation, large‐scale horizontal gradients in temperature, humidity and isotopic composition are neglected; that is, there are no large‐scale horizontal advective forcing terms. The large‐scale vertical velocity *ω*_*LS*_ has a cubic shape so as to reach its maximum (in absolute value) *ω*_*LSmax*_ at a pressure *p*_*max*_ and to smoothly reach 0 at the surface and at 100 hPa (Bony et al., 2008). We set *p*_*max*_ = 500 hPa and *ω*_*LSmax*_ = −60, −20, or +20 hPa/day (Simulations “*ω*_*LS*_−60,” “*ω*_*LS*_−20” and “*ω*_*LS*_+20”).

It is observed that the amount effect is stronger when convection and large‐scale vertical velocity profiles peak higher in altitude (Lacour et al., 2017; Torri et al., 2017). We thus test different values for *p*_*max*_, from 400 hPa to 600 hPa (Simulations “*p*400” and “*p*600”).

To compare the isotopic response to precipitation changes associated with large‐scale circulation to those associated with SST changes, we also test SST values of 26°C and 33°C (Simulations “26C” and “33C”).

To check the robustness of our results to horizontal resolution, we perform a simulation identical to control, except for 200 m horizontal resolution and a horizontal domain size of 25.6km × 25.6 km (Simulation “200m”).

**Figure 1:**
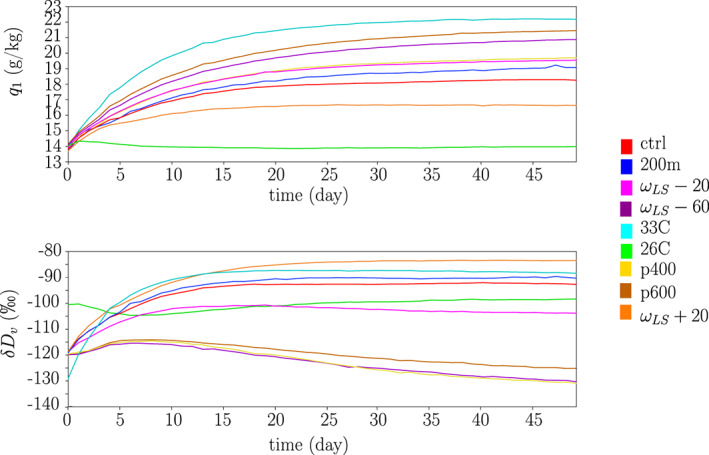
Temporal evolution of the water vapor mass mixing ratio *q* (top) and of the water vapor *δD* (bottom) in the lowest layer during 50 days of simulations, for the different simulations.

Figure 1 shows the temporal evolution of daily mean water vapor mass mixing ratio *q* and water vapor *δD* (*δD*_*v*_) in the lowest layer for the different simulations. Variables evolve from their initial state to a steady‐state value that depends on each simulation. In most simulations it takes about 20 days to reach the steady state, but it takes longer for *δD* values in case of large‐scale ascent, especially in the *p*400 simulation. Therefore, all simulations are run for 50 days, except for the *p*400 simulation that is run for 75 days. The last 10 days are analyzed.

### 2.3 Spatial Organization of Convective Features

**Figure 2:**
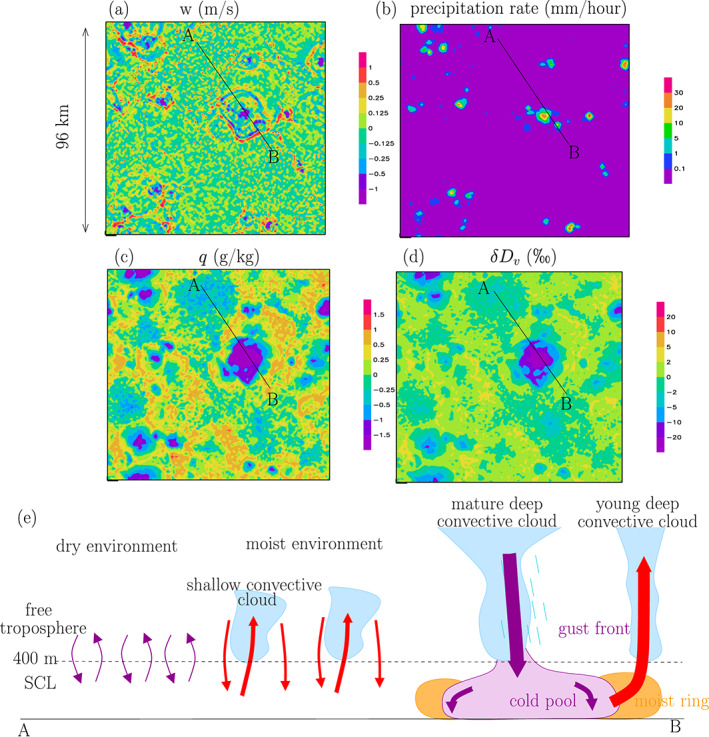
Maps of vertical velocity anomalies (a), precipitation rate (b), water vapor mass mixing ratio anomalies (c), and water vapor *δD* anomalies (d), at the lowest model level, at the last output time of the simulation, for the ctrl simulation. Anomalies are relative to the domain average. (e) Schematic to identify the different convective features along the A‐B transect. Red arrows indicate moist and enriched drafts, whereas purple arrows indicate dry and depleted drafts. Large arrows indicate strong drafts, whereas small arrows indicate weak drafts.

Figures 2a–2d show snapshots of the vertical velocity *w* at the SCL top, precipitation rate at the surface, *q* and *δD*_*v*_ anomalies relative to the domain mean near the surface, for the control simulation. This is the opportunity to illustrate the different convective features that will be discussed throughout the paper (Figure 2e). A video of the temporal evolution of these variables is available in supporting information Movie S1.

The most salient feature is the big dry and depleted patch near the center of the domain, corresponding to a mature deep convective system. At its center, the rain rate is strong and there is a strong convective downdraft, driven by the evaporation of rain drops (Zipser, 1969). The downdraft spreads near the surface like a density current, forming the cold pool. The deep convective system is surrounded by a thin line of descending air, corresponding to the gust fronts of the cold pool. The cold pool edges are surrounded by moist and strongly ascending air, called the moist rings (Torri & Kuang, 2016b). These moist rings can give rise to new convective cells (Torri et al., 2015). Several deep convective systems, at different stages, can be present across the domain.

Outside these convective systems, the environment is animated by shallow convection, as shown by the juxtaposition of ascending and descending spots. Some regions of the environment are relatively moist and covered by shallow convective clouds. Other regions are relatively dry, cloud‐free, with dry shallow convection. They may be associated with slow clear‐sky radiatively driven descent (Bretherton et al., 2005).

This spatial organization of convective features looks qualitatively similar for all simulations, except that convective systems are more numerous in case of large‐scale ascent and more sparse in case of large‐scale descent (Movie S2).

Being able to simulate all these features motivates the use of the high horizontal resolution of 750 m.

### 2.4 Sensitivity to SST and Large‐Scale Vertical Velocity

**Figure 3:**
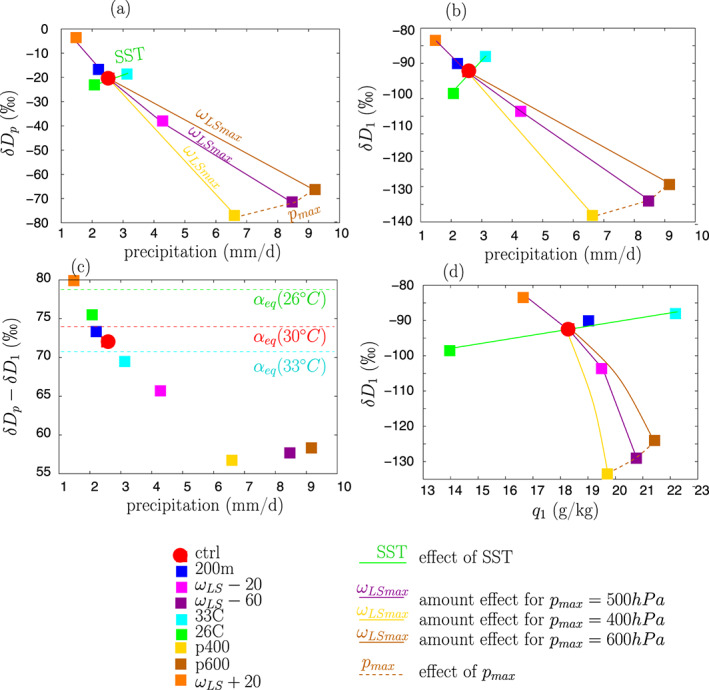
(a) Precipitation *δD* as a function of precipitation rate simulated by SAM in the different simulations (listed in Table 1). (b) Same as (a) but for water vapor *δD* in the lowest layer. (c) Same as (a) but for *δD*_*p*_ − *δD*_*v*_. The dashed cyan, red, and green lines show the expected *δD*_*p*_ − *δD*_*v*_ values if rain and water vapor were in isotopic equilibrium at 26°C, 30°C, and 33°C, respectively. These values are calculated as (*α*_*eq*_(*SST*) − 1)·1,000. When a marker is above the dashed line, the rain is more enriched than if in equilibrium with the vapor. Conversely, when a marker is below the dashed line, the rain is more depleted than if in equilibrium with the vapor. (d) Same as (b) but as a function of water vapor mass mixing ratio in the lowest layer. The results from SAM as in (d) are indicated again as empty squares for comparison.

When *ω*_*LSmax*_ becomes more negative, precipitation increases and precipitation *δD* (*δD*_*p*_) decreases (Figure 3a, purple line), consistent with the amount effect (Dansgaard, 1964). In contrast, when SST increases, precipitation increases slightly and *δD*_*p*_ increases (Figure 3a, green line), opposite to the amount effect. This behavior was already noticed in single column versions of GCMs (Bony et al., 2008) and means that *δD*_*p*_ responds differently to precipitation changes, depending on whether these changes are dynamical (mediated by the large‐scale circulation) or thermodynamical (mediated by SST). This is also consistent with the vertically integrated view of the amount effect in which large‐scale convergence is responsible for the *δD*_*p*_ variations (Bailey et al., 2017; Lee et al., 2007; Moore et al., 2014).

Note that independently prescribing SST and *ω*_*LS*_ is quite artificial. In reality, the large‐scale circulation depends on the SST, with ascending motions favored over warmer SST (Bony et al., 2004; Sobel & Bretherton, 2000). If in our simulations the large‐scale circulation was allowed to adapt to different SST following the weak temperature gradient approach (Sobel & Bretherton, 2000), we would expect the results to be very similar to those with imposed *ω*_*LS*_, since the effect of *ω*_*LS*_ overwhelms that of SST (Bony et al., 2008).

The amount effect is the strongest; that is, the slope of *δD*_*p*_ as a function of precipitation rate is the steepest, when the profile of large‐scale vertical velocity peaks high in altitude (Figure 3a, yellow lines steeper than purple lines), consistent with satellite observations (Lacour et al., 2017).

The near‐surface water vapor *δD* (*δD*_*v*_) behaves in a way that is very similar to *δD*_*p*_ (Figure 3b). This is consistent with observations showing that the isotopic composition of the rain often varies in concert with that of the near‐surface vapor (Aemisegger et al., 2015; Graf et al., 2019; Kurita et al., 2011), and reflects the partial isotopic equilibration of the rain with the vapor as it falls (Lee & Fung, 2008). From *ω*_*LS*_ + 20 to *ω*_*LS*_ − 60, most of the decrease in *δD*_*p*_ (−67.8‰) is due to the decrease in *δD*_*v*_ (−45.6‰, i.e., 67% of the decrease in *δD*_*p*_). This confirms that understanding what controls the SCL water vapor composition is necessary and relevant to understanding what controls the precipitation composition.

Rain‐vapor exchanges also contribute to the amount effect. For example, from *ω*_*LS*_ + 20 to *ω*_*LS*_ − 60, they contribute to approximately one third of the decrease in *δD*_*p*_ (Figure 3c). In the control simulation and in case of large‐scale ascent, the precipitation is more depleted than if in equilibrium with the vapor (Figure 3c, red and purple markers below the red dashed line). This is consistent with rain falling from higher in altitude without fully equilibrating with the near‐surface vapor. In contrast, in the *ω*_*LS*_ + 20 simulation, the rain is more enriched than if in equilibrium with the vapor (Figure 3c, orange marker above the red dashed line). This reflects evaporative enrichment. Variable *δD*_*p*_ − *δD*_*v*_ increases as near‐surface relative humidity decreases, because rain drops evaporate more efficiently in a dry environment, and the evaporative enrichment is also more efficient for a given evaporated fraction in a dry environment. Since near‐surface relative humidity is strongly tied to precipitation rate in our simulations, *δD*_*p*_ − *δD*_*v*_ generally decreases as precipitation rate increases.

Variable *δD*_*v*_ as a function of *q* shows a similar behavior as when plotted as a function of precipitation (Figure 3d). When large‐scale ascent increases, *q* increases and *δD*_*v*_ decreases, consistent with the amount effect observed by satellite in the water vapor (Lacour et al., 2017; Worden et al., 2007). In contrast, when SST or the shape of large‐scale ascent (*p*_*max*_) vary, *q* and *δD*_*v*_ vary in a correlated manner.

The results are robust with respect to resolution: The results for Simulation 200m are very close to those for ctrl (Figure 3, blue vs. red).

To understand what controls *q* and *δD*_*v*_ across the different simulations, we now look at some domain mean vertical profiles and at the water budget of the SCL.

### 2.5 Vertical Profiles and Water Budget of the SCL

**Figure 4:**
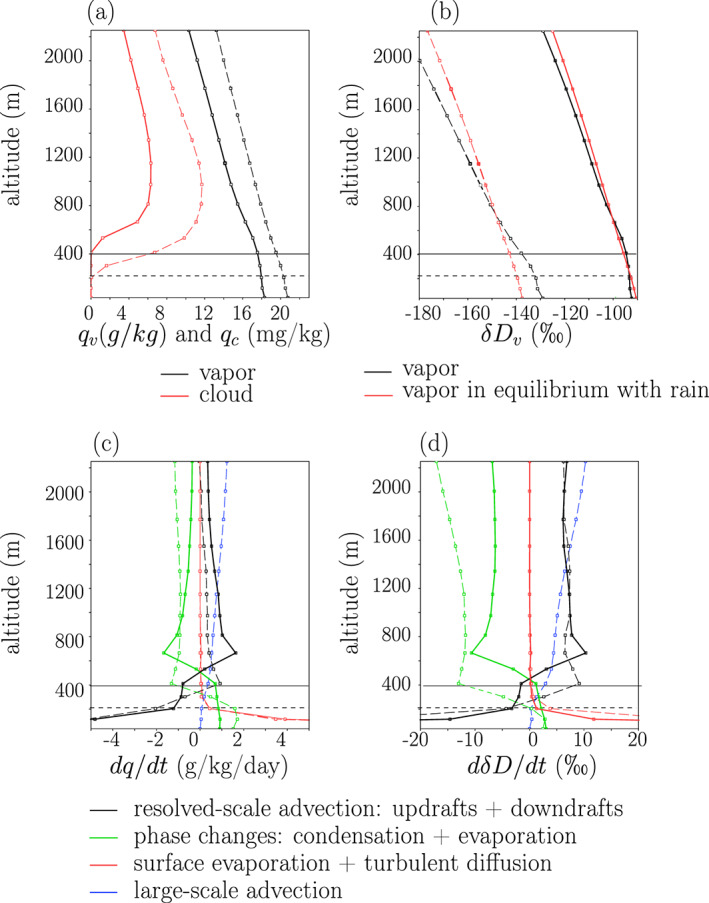
(a) vertical profiles of *q* (black) and cloud water (red) for the ctrl (solid) and *ω*_*LS*_ − 60 simulations (dashed). (b) Same as (a) but for water vapor *δD* (black) and the water vapor *δD* that would be in equilibrium with the rain (red). If the red line is more negative than the black line, the vapor is more enriched than if in equilibrium with the rain, which is equivalent to the rain being more depleted than if in equilibrium with the vapor. (c, d) Vertical profiles of tendencies in water vapor mass mixing ratio (c) and in water vapor *δD* (d) due to resolved‐scale advection (i.e., explicitly simulated updrafts and downdrafts) (black), large‐scale advection (blue), surface evaporation and subgrid‐scale diffusion (red), and phase changes (green) for the ctrl (solid) and *ω*_*LS*_ − 60 simulations (dashed). All results are averaged over 10 days. The horizontal solid and dashed black lines represent the SCL top for the ctrl (solid) and *ω*_*LS*_ − 60 simulations (dashed).

We define the SCL top, *z*_*T*_, as the highest level where the cloud fraction is smaller than 1%. Other definitions were also tested (e.g., highest level where the cloud water content is smaller the 10% of its maximum value), giving the exact same results. The SCL top varies from level 5 (411.6 m) in ctrl to level 3 (204.8 m) in *ω*_*LS*_−60 (last column of Table 1). It is lower when the SCL is more moist in terms of relative humidity, which is the case when there is large‐scale ascent. The cloud water content is near zero in the SCL and rises abruptly above it (Figure 4a, red).

Humidity and *δD*_*v*_ decrease with altitude. This is consistent with the preferential removal of heavy isotopes during condensation. Within the SCL, *q* and *δD* are relatively constant (Figures 4a and 4b, black): in ctrl, the vertical gradients in *q* and in *δD*_*v*_ within the SCL are respectively −2.0 g/kg/km and −6.0‰/km, whereas they are more than twice and 3 times larger, respectively, within the next 500 m above the SCL. This shows that the SCL is relatively well mixed in the vertical (Betts & Ridgway, 1989; De Roode et al., 2016; Stevens, 2006).

In Figure 4b, we can see that *δD*_*v*_ (black) is within a few ‰ of the value for the vapor in equilibrium with the rain (red). This is consistent with progressive equilibration of the rain with the surrounding vapor as it falls (Lee & Fung, 2008). When looking in more detail, we can see that above 800 m, the rain is slightly more enriched than if in equilibrium with the vapor, because the rain forms in updrafts that are generally more enriched than the domain mean water vapor. Then, as rain falls, it partially, but not fully, equilibrates with the vapor, so it keeps some of its depleted isotopic composition from higher altitudes. It thus becomes more depleted than if in equilibrium with the vapor, especially in case of large‐scale ascent (red dashed line more negative than the black dashed line). Finally in the SCL, the rain gets enriched by evaporation, especially if the rain rate is weak as in the ctrl simulation (red solid line less negative than the black solid line in the SCL).

Figure 4c shows the humidity tendencies associated with different processes, which are directly available in model outputs. Table 2 summarizes these tendencies as vertical integrals over the SCL. In the SCL, the air is mainly moistened by surface evaporation and turbulent diffusion (red) and is dehydrated by resolved‐scale advection, that is, updrafts and downdrafts that are explicitly simulated by SAM (black). Rain evaporation is estimated as the water vapor tendency associated with phase changes (green), at levels where this tendency is positive. It has a slightly moistening effect. Large‐scale advection, when present, has a very small effect in the SCL (blue, Table 2).

**Table 2:** Terms of the Vertically Integrated Water Budget of the SCL, in mm/day of Water Vapor: Surface Evaporation, Rain Evaporation, Resolved‐Scale Advection (i.e., Updrafts and Downdrafts That Are Explicitly Simulated by SAM), and Large‐Scale Advection

	Surface	Rain	Resolved‐scale	Large‐scale		
	evaporation	evaporation	advection	advection		
Name	(mm/day)	(mm/day)	(mm/day)	(mm/day)	*a*_*u*_ (%)	$\bar{f_{u}}/\bar{f}$ (%)
ctrl	2.61	0.44	−3.04	0	48	79
*ω*_*LS*_−60	1.86	0.47	−2.33	0.01	47	74
*ω*_*LS*_−20	2.26	0.45	−2.72	0.01	47	76
*ω*_*LS*_+20	3.18	0.44	−3.56	−0.01	49	87
26C	2.16	0.38	−2.54	0	49	91
33C	3.09	0.51	−3.58	0	48	83
*p*400	2.27	0.58	−2.84	0.00	48	80
*p*600	1.64	0.46	−2.19	0.02	48	75
200m	2.23	0.32	−2.51	0	48	85

*Note*. They correspond to the vertical integral of the curves on Figure 4c. The sum of the four terms should be null (small deviation may reflect rounding approximations). The sixth column indicates the fraction *a*_*u*_ of the area that is covered by updrafts. In Figure 5a, it corresponds to the fraction of the area under the curve that is on the right of the vertical dashed line. The seventh column indicates the fraction $\bar{f_{u}}/\bar{f}$ of the total transport of water out of the SCL that is done by updrafts. It is calculated as $\int_{w=0}^{+\infty }\frac{f(w)}{dw}\cdot dw/\int_{w=-\infty }^{+\infty }\frac{f(w)}{dw}\cdot dw$, where *f* (*w*) is the water flux in each *w* bin. In Figure 5c, it corresponds to the fraction of the area under the curve that is on the right of the vertical dashed line.

In the free troposphere, the air is moistened by resolved‐scale advection (black) and dehydrated by cloud condensation (green) (Figure 4c). When present, the tendency associated with large‐scale advection becomes the main moistening term (dashed blue). It is compensated by condensation, and thus explains the larger precipitation rate in case of large‐scale ascent (Skyllingstad & de Szoeke, 2015).

The *δD*_*v*_ tendencies show a behavior that is very similar to humidity tendencies: processes that moisten the air enrich the water vapor, and processes that dehydrate the air deplete the water vapor (Figure 4d). We note that rain evaporation has an enriching effect, contrary to what we would expect if a very small fraction of rain drops evaporate (Worden et al., 2007) and more consistent with what we would expect if a significant portion of rain drops evaporate (Risi, Bony, Vimeux, Chong, et al. 2010; Tremoy et al., 2014).

To summarize, the resolved‐scale advection is a crucial component of the SCL water budget, since it compensates for the moistening by surface and rain evaporation. The transport of water by the resolved‐scale advection can be written as $\bar{w^{\prime }q^{\prime }}$, where *w′* and *q′* are anomalies in *w* and *q* relative to the domain mean. The transport of water thus reflects the correlation between *w′* and *q′*. The net export of moisture out of the SCL implies that *w′* and *q′* correlate (i.e., updrafts are generally more moist and downdrafts are generally drier) at *z*_*T*_. But the efficiency of updrafts and downdrafts to dry the SCL depends on the how much *w′* and *q′* correlate. Similarly, the efficiency of updrafts and downdrafts to deplete the SCL depends on how much (*wq*)*′* and $\delta D_{v}^{\prime }$ correlate. Therefore, we now look at air properties as a function of *w*.

### 2.6 Properties of Downdrafts and Updrafts

To document air properties as a function of velocity *w*, we bin *q* and *δD* into 14 categories corresponding to intervals of *w* at *z*_*T*_ in m/s: ]−*∞*; −2[, [−2; −1], ]−1; −0.5], ]−0.5; −0.25], ]−0.25, −0.125], ]−0.125; −0.0625], ]−0.0625; 0, ]0; 0.0625], ]0.0625; 0.125], ]0.125; 0.25], ]0.25; 0.5], ]0.5; 1], ]1; 2] and ]2;+*∞*[. We consider the full spectrum of *w* values rather than just binning into convective updrafts, convective downdrafts and weak drafts in the environment as in Thayer‐Calder and Randall (2015), because otherwise the results would be too sensitive to the arbitrary definition of the environment (Torri & Kuang, 2016a). In addition, the diversity of convective features identified in section 2.3, such as convective downdrafts, gust front, moist rings or shallow convective drafts, are more likely to be represented by the full spectrum of *w* values.

**Figure 5:**
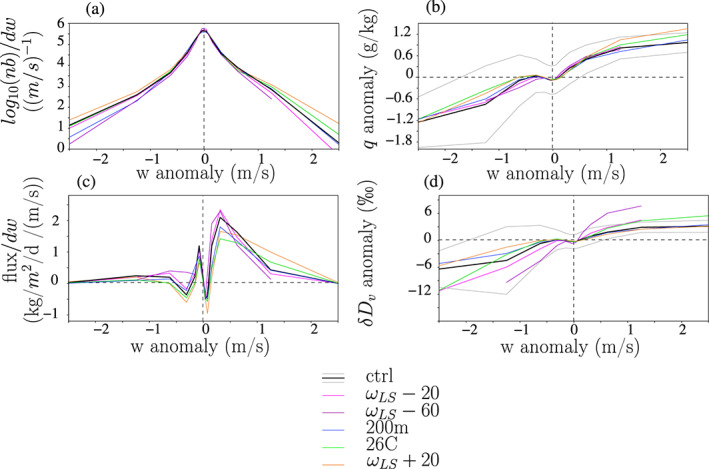
Draft properties binned as a function of instantaneous vertical velocity anomaly at *z*_*T*_ (*w*), for the different simulations. All properties are calculated at the SCL top. (a) Number of grid points (*nb*) in each bin, in logarithmic scale. Values are normalized by the bin width for an easier visual integration: The area under the curve, calculated as $\int_{w=-\infty }^{+\infty }\frac{nb(w)}{dw}\cdot dw$, equals the total number of locations and snapshots, that is, 128 × 128 grid points × 10 snapshots. (b) Water vapor mass mixing ratio anomaly at *z*_*T*_ relative to the domain mean. The gray line shows the mean ± the standard deviation for the ctrl simulation. (c) Water flux through the SCL top. Values are normalized by the bin width for an easier visual integration: The area under the curve, calculated as $\int_{w=-\infty }^{+\infty }\frac{f(w)}{dw}\cdot dw$, where *f* (*w*) is the water flux in each *w* bin, equals the domain mean $\rho (z_{T})\cdot \bar{w^{\prime }q^{\prime }}$. Positive values indicate export of water out of the SCL, whereas negative values indicate import of water into the SCL. (d) *δD*_*v*_ anomaly at *z*_*T*_ relative to the domain mean. Only bins where there are more than 10 points are shown. For a clearer plot, Simulations *p*400 and *p*600 are omitted because they give results similar to *ω* − 60, and Simulation 33C is omitted because it gives results similar to ctrl.

The probability distribution of *w* peaks at 0 m/s (Figure 5a). The distribution is nearly symmetrical around 0 m/s, with the fraction of updrafts covering 47% to 49% of the domain (Table 2, sixth column).

Strong downdrafts (*w* <−1 m/s) are generally drier relative to the domain mean (Figure 5b), consistent with previous studies (Zuidema et al., 2017). They correspond to the center of convective systems or to the gust front of cold pools (Figure 2). We notice however that moderate downdrafts around −0.5 m/s are slightly more moist than the domain mean. They correspond to downdrafts in the moist and shallow convective regions of the domain (Figure 2e).

Updrafts are generally moist relative to the domain mean (Figure 5c). This is also expected from SCL water balance (section 3.1.5) and is consistent with previous studies (Cruette et al., 2000; Kuang & Bretherton, 2006). Updrafts often originate from moist rings (Figure 2), whose anomalous humidity may come from enhanced surface evaporation (Langhans & Romps, 2015) or from rain evaporation (Torri & Kuang, 2016b).

The contribution of a given *w* bin to the total flux of water through the SCL top, expressed in kg/m^2^/s, can be calculated as

$$f(w)\;=\;P(x,y,t\in W)\cdot \frac{\sum_{x,y,t\in W}\rho (z_{T})\cdot w(x,y,z_{T},t)\cdot \left(q(x,y,z_{T},t)\;-\;\bar{q}(z_{T})\right)}{\sum_{x,y,t\in W}1}$$

where *W* is the ensemble of grid points and time steps *x*, *y*, *t* for which the vertical velocity *w*(*x*, *y*, *z*_*T*_, *t*) falls into the *w* bin, *P*(*x*, *y*, *t*∈*W*) is the fraction of grid points and time steps that fall in the *w* bin, *ρ*(*z*) is the density assumed to be an unique function of altitude, *w*(*x*, *y*, *z*_*T*_, *t*) and *q*(*x*, *y*, *z*_*T*_, *t*) are the grid‐scale vertical velocity and water vapor mass mixing ratio at SCL top, and $\bar{q}(z_{T})$ is the domain mean, time mean water vapor mass mixing ratio at SCL top. The contribution of a given *w* bin to the flux is thus strong if the fraction of grid points and time steps that fall in the *w* bin is high, if *w* is high, and if the *q* anomalies are high and positively correlated with *w*. As a consequence, weak updrafts and downdrafts contribute relatively little to the water flux out of the SCL, because their velocity and humidity anomalies are small. Strong downdrafts and updrafts also contribute little to the water flux out of the SCL (Figure 5c), because their probability of occurrence is too small (Figure 5a). The small contribution from strong drafts is consistent with the major contribution of the environment to the moist static energy budget (Thayer‐Calder & Randall, 2015; Torri & Kuang, 2016a). The largest contribution to the water flux actually comes from moderate updrafts around 0.5 m/s (Figure 5c). They correspond mainly to updrafts in regions of the environment that are relatively moist and animated by shallow convection (Figure 2). In contrast, moderate downdrafts contribute negatively to the water flux (Figure 5c), because of their slightly positive *q* anomalies (Figure 5b).

All in all, although updrafts cover about half of the domain, they contribute more than downdrafts to the water export out of the SCL: between 74% and 91% depending on simulations (Table 2, last column). In the SI, we show that the relative contribution of the updrafts to the water export out of the SCL depends crucially on the correlations between *q* and *w* for updrafts and for downdrafts (Text S1). For updrafts this correlation is strong, but for downdrafts it is disrupted by the presence of moderate downdrafts in moist shallow convective regions (Figures 2 and 5b). This is why updrafts contribute more to the water export out of the SCL than downdrafts.

All simulations exhibit qualitatively similar features as described above. Results are very similar in ctrl and in 200m (Figure 5 blue vs. black), showing that the horizontal resolution of our simulations is sufficient to capture updraft and downdraft properties.

There is a tight relationship between *q* and *δD*_*v*_: The distribution of *δD*_*v*_ as a function of *w* echoes that of *q* (Figure 5d). In case of large‐scale ascent however, *δD*_*v*_ anomalies are more positive in updrafts and more negative in downdrafts (Figure 5d purple, Movies S1 and S2). They cannot be fully explained by anomalies in *q*, which are similar to those in ctrl. What controls the strength of the *q* and *δD*_*v*_ anomalies as a function of *w*? In the next section, we check the hypothesis that these arise from vertical gradients.

### 2.7 Link With Vertical Gradients

**Figure 6:**
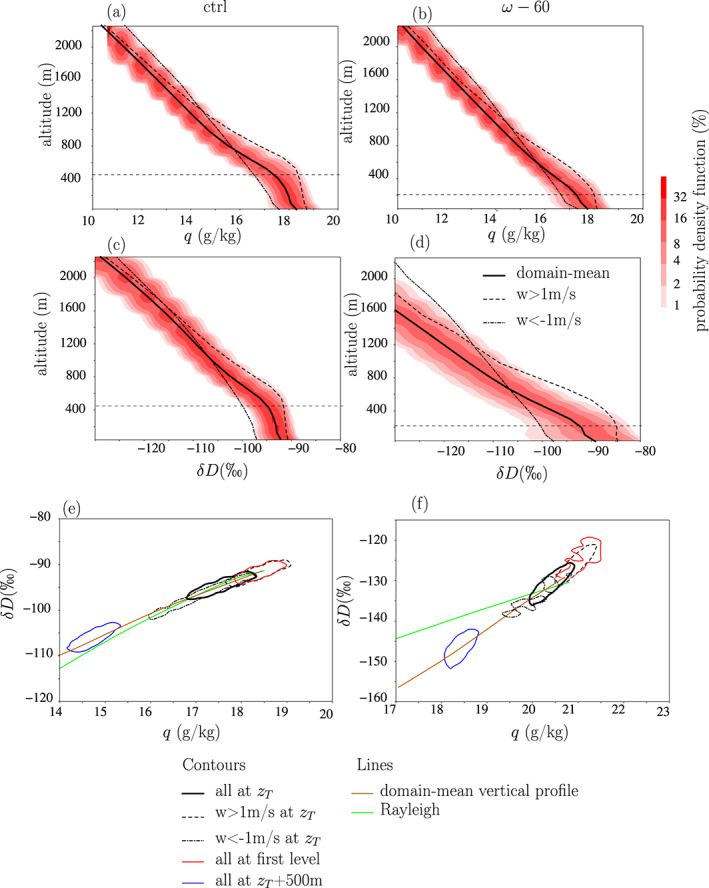
(a) Probability density function of water vapor mass mixing ratio as a function of altitude in the ctrl simulation. The domain mean profile is also shown in solid black, the mean profile for updrafts in dashed black, and the mean profile for downdrafts in dash‐dotted black. The horizontal dashed line corresponds to the SCL top. (b) Same as (a) but for the *ω*_*LS*_ − 60 simulation. (c) Same as (a) but for *δD*_*v*_ profiles. (d) Same as (c) but for the *ω*_*LS*_ − 60 simulation. (e) Joint *q* − *δD*_*v*_ distribution for all locations in the domain at altitude *z*_*T*_ (solid black), all updrafts at altitude *z*_*T*_ (dashed black), all downdrafts at altitude *z*_*T*_ (dash‐dotted black), all locations in the domain near the surface (red), and all locations at altitude *z*_*T*_ + 500 m (blue). The contour indicates a probability density function of 2%. The domain mean vertical profile is also shown as a brown line. The green line represents what we would expect if the domain mean vertical *δD*_*v*_ profile followed a Rayleigh line with fractionation coefficients calculated as a function of domain mean temperature. (f) Same as (e) but for the *ω*_*LS*_ − 60 simulation.

As an illustrative example, we compare in Figure 6 the properties of strong updrafts at *z*_*T*_ (*w*(*x*, *y*, *z*_*T*_, *t*)>1 m/s, dashed black), strong downdrafts at *z*_*T*_ (*w*(*x*, *y*, *z*_*T*_, *t*)<−1 m/s, dash‐dotted black) and the domain mean (thick black). Here we look at strong drafts because although they contribute little to the total water export out of the SCL, their strong *q* and *δD*_*v*_ anomalies allows us to plot clearer figures. The mechanisms that we will identify can then be generalized to weaker drafts.

Above 1000 m, the downdrafts are located in the air that is more moist than the domain mean, because they usually stem from updrafts in convective systems (Figure 6a, dash‐dotted black). But as they descend, they keep their low *q* from higher altitudes. Hence, they are drier than the domain mean when they arrive at *z*_*T*_. If we assumed that *q* was conserved during descent, we would find that downdrafts at *z*_*T*_ originate on average from just a few tens of meters above *z*_*T*_, consistent with previous numerical modeling (Torri & Kuang, 2016a), conceptual modeling (Betts, 1976) and observational studies (de Szoeke et al., 2017; Kingsmill & Houze, 1999; Schiro & Neelin, 2018; Zipser, 1969) showing that downdrafts are shallow.

In strong updrafts, the air is moist relative to the domain mean (Figure 6a, dashed black). This can be explained by two reasons. First, strong updrafts stem from locations that are the moistest near the surface. Second, they rise through the SCL with nearly constant *q*.

At *z*_*T*_, the water vapor is more enriched in updrafts, and more depleted in downdrafts, relative to the domain mean (Figure 6c). The behavior of *δD*_*v*_ echoes that of *q*. Figure 6e shows that near‐surface air (red), strong updrafts, strong downdrafts and air higher in altitude (blue) align in the *q* − *δD*_*v*_ diagram, following a Rayleigh line (Figure 6e, green line). This is consistent with the downdrafts being more depleted than the domain mean because they come from a higher altitude.

In case of large‐scale ascent, the vertical gradient in *δD*_*v*_ is much steeper (Figure 6d). Since the vertical gradient in *q* is almost the same, this is explained by a steeper *q* − *δD*_*v*_ relationship (Figure 6f). The *q* − *δD*_*v*_ relationship is about twice steeper than predicted by a Rayleigh distillation (Figure 6f, green). We hypothesize that the steeper *q* − *δD*_*v*_ relationships in the vertical (Figure 6f, brown), combined with *w* anomalies, leads to steeper *q* − *δD*_*v*_ relationships across updrafts and downdrafts (Figure 6f). Therefore, *δD* *_v_* anomalies in updrafts and downdrafts relative to the domain‐mean are much stronger than in ctrl (Figure 5, green, and Figure 2f), even though the moisture anomalies in updrafts and downdrafts are similar to those in ctrl.

Now we test the hypothesis that the steepness of the domain mean *q* − *δD*_*v*_ relationship in the vertical controls the steepness of the *q* − *δD*_*v*_ relationship across updrafts and downdrafts. The steepness of the domain mean *q* − *δD*_*v*_ relationship in the vertical can quantified as

(1) $$\alpha_{z}\;=\;1+\frac{ln\left(\bar{R_{v}}(z_{T}+500m)/\bar{R_{v}}(z_{T})\right)}{ln\left(\bar{q}(z_{T}+500m)/\bar{q}(z_{T})\right)}$$

where $\bar{R_{v}}$ and $\bar{q}$ are the domain mean isotopic ratio and water vapor mass mixing ratio. Parameter *α*_*z*_ is an effective fractionation coefficient that represents the fractionation coefficient that would be needed for a Rayleigh distillation to fit the simulated domain mean *δD*_*v*_ profiles.

Similarly, the steepness of the *q* − *δD*_*v*_ relationships between updrafts and the domain mean, and between downdrafts and the domain mean, can be quantified as

(2) $$\alpha_{u}\;=\;1+\frac{ln(R_{u}/R_{1})}{ln(q_{u}/q_{1})}$$

(3) $$\alpha_{d}\;=\;1+\frac{ln(R_{d}/R_{1})}{ln(q_{d}/q_{1})}$$

where *α*_*u*_ and *α*_*d*_ are effective fractionation coefficients for updrafts and downdrafts, *q*_*u*_, *q*_*d*_, *R*_*u*_, and *R*_*d*_ are the water vapor mass mixing ratio and isotopic ratios effectively transported by updrafts and downdrafts through the SCL top (the exact calculation of *q*_*u*_, *q*_*d*_, *R*_*u*_, and *R*_*d*_ will be better detailed in section 3.3), and *q*_1_ and *R*_1_ are the water vapor mass mixing ratio and isotopic ratio near the surface in average over the domain.

**Figure 7:**
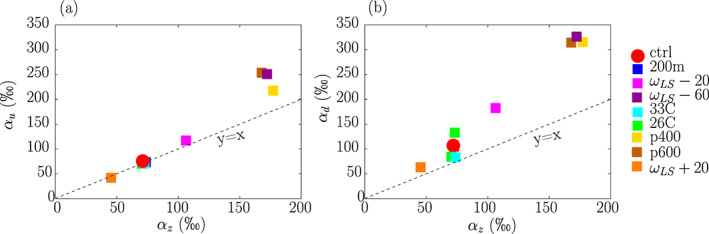
(a) Effective fractionation coefficient in updrafts (*α*_*u*_) as a function of the effective fractionation coefficient for the domain mean vertical profile *α*_*z*_, calculated following Equation 1. All fractionation coefficients are expressed in ‰. (b) Same for the effective fractionation coefficient in downdrafts (*α*_*d*_).

Simulations with higher *α*_*z*_ values also have higher *α*_*u*_ and *α*_*d*_ values (Figure 7). This confirms that the steepness of the *q* − *δD*_*v*_ relationship between updrafts and the domain mean, and between downdrafts and the domain mean, reflects the steepness of the *q* − *δD*_*v*_ relationship in the vertical.

We have analyzed in detail the properties of the different drafts. But now, what is the quantitative effect of these drafts on the SCL *q* and *δD*? To address this question, we now develop an analytical model for the SCL that emulates the LES simulations but allows us to disentangle and quantify the different effects.

## 3 Analytical Model for the SCL

Based on the analysis of the LES simulations in the previous section, we design an analytical model for the SCL. It is inspired by the SCL water budget presented in Risi et al. (2019), itself inspired by (Benetti et al., 2014, 2015). It is an extension of the closure equation by Merlivat and Jouzel (1979).

### 3.1 Analytical Model Equations

#### 3.1.1 Budget Equations

**Figure 8:**
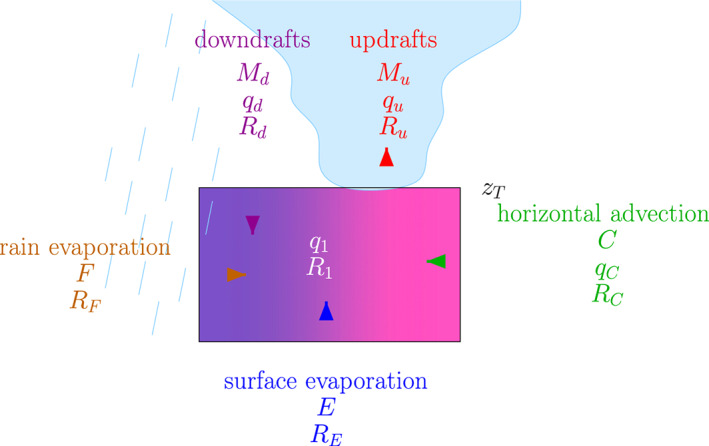
Schematics showing the simple box model on which the analytical model is based and illustrating the main notations. The shades in the SCL illustrate that updrafts preferentially occur where the air is moist and the water vapor is enriched, whereas downdrafts preferentially occur where the air is dry and the water vapor is depleted.

We consider a simple box representing the SCL (Figure 8). We showed in section 2.5 that the SCL was relatively well mixed in the vertical. Therefore, we assume that the SCL is vertically well mixed for water vapor mass mixing ratio *q* and for water vapor *δD*, which we, respectively, denote as *q*_1_ and *δD*_1_. We also assume that the SCL is at steady state: *q*_1_, *δD*_1_, and its depth do not vary with time.

The air mass budget of the SCL involves the mass flux entering the SCL from above, *M*_*d*_ (positive downward), the mass flux entering the SCL through convergence of air, *C* (positive or negative), and the mass flux leaving the SCL through its top, *M*_*u*_ (positive upward):

(4) $$M_{u}\;=\;M_{d}+C$$

Since SAM uses a double‐periodic domain, mass conservation implies that *M*_*u*_ = *M*_*d*_. Here we retain *C* for generality.

Following the tendency analysis in section 2.5, the water budget of the SCL involves the air entering the SCL from above with humidity *q*_*d*_, the convergence of air into the SCL with humidity *q*_*C*_, the water arising from the surface evaporation flux *E*, the water arising from the rain evaporation flux *F*, and the air leaving the SCL through its top with humidity *q*_*u*_:

(5) $$M_{u}\cdot q_{u}\;=\;M_{d}\cdot q_{d}+C\cdot q_{c}+E+F$$

The isotopic budget of the SCL involves the water vapor entering the SCL from above with isotopic ratio *R*_*d*_, the water vapor entering the SCL through horizontal advection with isotopic ratio *R*_*C*_, the water arising from surface evaporation with isotopic ratio *R*_*E*_, the water arising from the rain evaporation with isotopic ratio *R*_*F*_, and the water vapor leaving the SCL through its top with isotopic ratio *R*_*u*_:

(6) $$M_{u}\cdot q_{u}\cdot R_{u}\;=\;M_{d}\cdot q_{d}\cdot R_{d}+C\cdot q_{c}\cdot R_{c}+E\cdot R_{E}+F\cdot R_{F}$$

#### 3.1.2 Surface Evaporation

For surface evaporation, we assume a bulk formula:

(7) $$E\;=\;c_{E}\cdot \left(q_{sat}^{surf}(SST)-q_{1}\right)$$

where $q_{sat}^{surf}(SST)\;=\;0.98\cdot q_{sat}(SST)$, *q*_*sat*_ is the humidity saturation as a function of temperature at the sea level pressure and *c*_*E*_ is a coefficient that includes the effect of the drag parameter and of the surface wind speed. The 0.98 factor represents the effect of salt on the saturated vapor pressure of water at the ocean surface (Zeng et al., 1998).

During evaporation, there are two kinds of isotopic fractionation. First, because of the difference of molar mass between H_2_O and *HDO*, at equilibrium *HDO* is more concentrated in the liquid phase than in the vapor. This effect is represented by an equilibrium fractionation coefficient *α*_*eq*_, which is a function of temperature (Majoube, 1971). Second, because of the difference of molecular diffusivity between H_2_O and *HDO*, before reaching equilibrium *HDO* evaporates more slowly than H_2_O. This effect is represented by a kinetic fractionation coefficient *α*_*K*_ (Merlivat & Jouzel, 1979). The isotopic composition of the surface evaporation *R*_*E*_ is assumed to follow Craig and Gordon's (1965) equation:

(8) $$R_{E}\;=\;\frac{R_{oce}/\alpha_{eq}(SST)-h_{1}\cdot R_{1}}{\alpha_{K}\cdot (1-h_{1})}$$

where *R*_*oce*_ is the isotopic ratio in the surface ocean water and *h*_1_ is the relative humidity normalized at the SST:

$$h_{1}\;=\;\frac{q_{1}}{q_{sat}^{surf}(SST)}$$

#### 3.1.3 Rain Evaporation

To calculate *R*_*F*_, we make many approximations. First, we showed in section 2.5 that the isotopic composition of the rain was close to the equilibrium with the SCL water vapor. Therefore, we assume that the isotopic composition of the rain at the SCL top is in equilibrium with the SCL water vapor. Second, we assume that rain drops evaporate like one drop in a homogeneous environment, following Stewart (1975)'s equation. Given these assumptions, we do not expected to exactly mimic the behavior of SAM. However, as will be shown in the following, the effect of rain evaporation is relatively small, so the results are not crucially sensitive to these assumptions. Our aim here is just to include this effect in a simple analytical way.

Detailed calculations are available in Text S2 and yield

$$R_{F}\;=\;A\cdot R_{1}$$

with

$$A\;=\;\frac{\alpha_{eq}(T(z_{T}))\cdot \left(1-{\left(1-f_{ev}\right)}^{\beta +1}\right)-\gamma \cdot \left(1-f_{ev}\right)\cdot \left(1-{\left(1-f_{ev}\right)}^{\beta }\right)}{f_{ev}}$$

where $\beta \;=\;\frac{1-\alpha_{eq}(T(z_{T}))\cdot \alpha_{Kev}\cdot (1-h_{ev})}{\alpha_{eq}(T(z_{T}))\cdot \alpha_{Kev}\cdot (1-h_{ev})}$, $\gamma \;=\;\frac{\alpha_{eq}(T(z_{T}))\cdot h_{ev}}{1-\alpha_{eq}(T(z_{T}))\cdot \alpha_{Kev}\cdot (1-h_{ev})}$, $h_{ev}\;=\;\frac{q_{1}}{q_{sat}(T_{1})}$ is the relative humidity, and *α*_*Kev*_ is the kinetic fractionation coefficient for rain drop evaporation. This coefficient reflects the relative diffusivities of the different isotopes, and is different from *α*_*K*_ used at the sea surface because the diffusive conditions are different (Mathieu & Bariac, 1996).

When *f*_*ev*_→0, *A*→1/*α*_*Kev*_, consistent with the evaporation of a very small portion of the rain drops. When *f*_*ev*_→1, *A*→*α*_*eq*_(*T*(*z*_*T*_)), consistent with the total evaporation of rain drops that were in equilibrium with the vapor. In between, *A* increases with *f*_*ev*_ and reaches *α*_*eq*_(*T*(*z*_*T*_)) at a faster rate when *h*_*ev*_ is closer to 1: When *h*_*ev*_→1, *A*→*α*_*eq*_(*T*(*z*_*T*_)) whatever *f*_*ev*_.

#### 3.1.4 Horizontal Advection

We neglect the effect of horizontal gradients in *q* and water vapor *δD*, consistent with the setup of the large‐scale forcing in SAM (section 2.2). The effect of horizontal advection was shown in a GCM to be secondary over tropical oceans, except near some coastal regions or in the subtropics where cold and dry air may arrive from regions with colder SSTs (Risi et al., 2019). In addition, the contribution to the water budget of the large‐scale convergence flux *C* associated with the large‐scale vertical velocity forcing is very small (Table 2).

Therefore, for simplicity we just set *q*_*C*_ = *q*_1_ and *R*_*C*_ = *R*_1_.

#### 3.1.5 Downdrafts and Updrafts

For the purpose of simplicity when deriving the equations, we consider only two categories of drafts (updrafts and downdrafts), but the equations can be extended to consider any number of draft categories (Text S3).

We showed in section 2.6 that updrafts were more moist and more enriched relative to the domain mean, whereas downdrafts were drier and more depleted relative to the domain mean. We define *r*_*u*_ and *r*_*d*_ as

(9) $$r_{u}\;=\;q_{u}/q_{1}$$

(10) $$r_{d}\;=\;q_{d}/q_{1}$$

We showed that the isotopic ratio in updrafts and downdrafts was strongly tied to their water vapor mass mixing ratio (section 2.6). For mathematical convenience, we assume that *R*_*u*_ and *R*_*d*_ are related to *q*_*u*_ and *q*_*d*_, respectively, through power law relationships (Risi et al., 2019):

(11) $$R_{u}\;=\;R_{1}\cdot r_{u}^{\alpha_{u}-1}$$

(12) $$R_{d}\;=\;R_{1}\cdot r_{d}^{\alpha_{d}-1}$$

where *α*_*u*_ and *α*_*d*_ represent the steepness of the *q* − *δD*_*v*_ relationship between updrafts and the domain mean and between downdrafts and the domain mean, as was already defined in Equations 2 and 3. Parameters *α*_*u*_ and *α*_*d*_ can be seen as effective fractionation coefficients. But in contrast to Rayleigh distillation, here *α*_*u*_ and *α*_*d*_ may take a broad range of possible values, reflecting a wide diversity of processes, including rain evaporation (Risi et al., 2008) or mixing (Galewsky & Hurley, 2010; Galewsky & Rabanus 2016).

#### 3.1.6 Equations for *q*_1_ and *δD*_1_

The water budget (Equation 5), combined with Equations (7), (10), and 9, yields:

(13) $$q_{1}\;=\;\frac{q_{sat}^{surf}(SST)+F/c_{E}}{1+\left(1/c_{E}\right)\cdot \left(M_{u}\cdot \left(r_{u}-1\right)-M_{d}\cdot \left(r_{d}-1\right)\right)}$$

As expected, the SCL humidity *q*_1_ increases with $q_{sat}^{surf}(SST)$ and with the rain evaporation flux *F*, and decreases with *M*_*u*_ (consistent with the drying effect of mixing through the SCL top) (Bretherton et al., 1995), and decreases as *q* anomalies in updrafts and downdrafts relative to the domain main increase are stronger in absolute values (*r*_*u*_ increases and *r*_*d*_ decreases).

The isotopic budget (Equation 6) can be solved for *R*_1_:

(14) $$R_{1}\;=\;\frac{R_{oce}/\alpha_{eq}(SST)}{h_{1}+\alpha_{K}\cdot (1-h_{1})\cdot \left(\left(1+F/E\right)\cdot \frac{M_{u}\left(r_{u}^{\alpha_{u}}-1\right)-M_{d}\cdot \left(r_{d}^{\alpha_{d}}-1\right)}{M_{u}\left(r_{u}-1\right)-M_{d}\cdot \left(r_{d}-1\right)}-F\cdot A/E\right)}$$

Therefore, *R*_1_ is a function of *R*_*oce*_, of fractionation coefficients and of nine parameters that vary across the LES simulations: SST, *c*_*E*_, *F*, *M*_*u*_, *M*_*d*_, *r*_*u*_, *r*_*d*_, *α*_*u*_, and *α*_*d*_. Finally, *δD*_1_ is calculated from *R*_1_.

If we neglect the fact that updrafts are more moist, that is, if *r*_*u*_ = 1, we get equations that are equivalent to those in (Risi et al., 2019). If we further neglect the fact the downdrafts are more depleted and updrafts are more enriched (*α*_*d*_ = *α*_*u*_ = 1), we find the classical Merlivat and Jouzel's (1979) equation:

(15) $$R_{1}\;=\;\frac{R_{oce}}{\alpha_{eq}(SST)}\cdot \frac{1}{h_{1}+\alpha_{K}\cdot (1-h_{1})}$$

Finally, if we neglect the updraft and downdraft mass fluxes (*M*_*u*_ = *M*_*d*_ = 0 ), or if we neglect the fact that downdrafts are drier and updrafts more moist (*r*_*u*_ = *r*_*d*_ = 1), then we get a saturated SCL:

(16) $$q_{1}\;=\;q_{sat}^{surf}(SST)$$

Therefore, *h*_1_ = 1 and the water vapor is in equilibrium with the ocean surface:

(17) $$R_{1}\;=\;\frac{R_{oce}}{\alpha_{eq}(SST)}$$

### 3.2 Some Discussion of the Analytical Model Equations

Equation 14 is instructive on how downdrafts and updrafts are expected to affect the isotopic composition of the SCL water vapor *R*_1_.

First, in absence of large‐scale convergence in the SCL, which is nearly the case in all our simulations, *M*_*u*_ = *M*_*d*_ and thus the sensitivity of *R*_1_ to *M*_*u*_ and *M*_*d*_ disappears. This means that while *q*_1_ depends on the strength of updrafts and downdrafts, *R*_1_ does not, a counterintuitive result that was already shown in Risi et al. (2019).

Second, for *α*_*d*_>1, which is the case in all our simulations, *R*_1_ increases as *r*_*d*_ decreases. This means that if downdrafts come from higher in altitude (i.e., *r*_*d*_ decreases), they are more depleted but they are also drier. Therefore, they bring a small amount of depleted water vapor into the SCL, and thus they deplete the SCL water vapor in heavy isotopes less efficiently. This is another counterintuitive result that was already shown in Risi et al. (2019).

Third, downdrafts deplete the SCL water vapor all the more efficiently as the *q* − *δD*_*v*_ relationship between downdrafts and the domain mean is steep (i.e., *α*_*d*_ is large). Similarly, the efficiency of updrafts to deplete the SCL water vapor increases as *α*_*u*_ increases.

Fourth, the relative contribution of the updrafts to the drying of the SCL by all drafts can be approximated by $M_{u}\cdot \left(r_{u}-1\right)/\left(M_{u}\cdot \left(r_{u}-1\right)-M_{d}\cdot \left(r_{d}-1\right)\right)=\left(r_{u}-1\right)/\left(r_{u}-r_{d}\right)$. It corresponds to the relative contribution of the updrafts to the water export out of the SCL $\bar{f_{u}}/\bar{f}$ (section 2.6). Note that the relative contribution of the updrafts to the drying of the SCL and the relative contribution of the updrafts to the water export out of the SCL are not exactly equal due to nonlinear effects. Similarly, the relative contribution of the updrafts to the depletion of the SCL by all drafts can be approximated by $\left(r_{u}^{\alpha_{u}}-1\right)/\left(r_{u}^{\alpha_{u}}-r_{d}^{\alpha_{d}}\right)$. Since *α*_*u*_ and *α*_*d*_ are close to 1, we thus expect that the relative contribution of the updrafts to the drying of the SCL is similar to the relative contribution of the updrafts to the depletion of the SCL.

### 3.3 Diagnosing Analytical Model Parameters From SAM Simulations

All parameters in Equations 13 and 14 can be diagnosed from SAM simulations.

The SCL top *z*_*T*_ was defined in section 3.1.1. The total upward mass flux per unit of area (in kg/m^2^/s) is calculated as

(18) $$M_{u}\;=\;\frac{1}{n_{x}\cdot n_{y}\cdot n_{t}}\underset{x,y,t\in U}{\sum }\rho (z_{T})\cdot w(x,y,z_{T},t)$$

where *n*_*x*_,*n*_*y*_ are the number of points in the x and y dimensions, *n*_*t*_ is the number of snapshots taken into account (10, with one snapshot every day), *w*(*x*, *y*, *z*, *t*) is the grid‐scale vertical velocity, and *U* is the ensemble of (*x*, *y*, *t*) where and when *w*(*x*, *y*, *z*_*T*_, *t*)>0.

The total downdraft mass flux is calculated in the same way except that the sum is done only for *x*,*y*,*t*∈*D*, where *D* is the ensemble of (*x*, *y*, *t*) where and when *w*(*x*, *y*, *z*_*T*_, *t*) ≤ 0.

The water vapor tendency due to upward mass fluxes is calculated as

(19) $$M_{u}\cdot \left(q_{u}-q_{1}\right)\;=\;\frac{1}{n_{x}\cdot n_{y}\cdot n_{t}}\underset{x,y,t\in U}{\sum }\rho (z_{T})\cdot w(x,y,z_{T},t)\cdot \left(q(x,y,z_{T},t)\;-\;\bar{q}(z_{T})\right)$$

where the overline means the average over *x*, *y*, *t*. From this equation, *q*_*u*_ is deduced. Note that *q*_*u*_ is not exactly the mean humidity in updrafts. It is an average weighted by mass fluxes. Otherwise, $M_{u}\cdot \left(q_{u}-q_{1}\right)$ would underestimate the upward water mass flux (Siebesma & Cuijpers, 1995; Yano et al., 2004).

The water vapor tendency due to downward mass fluxes is calculated in the same way except that the sum is done only where and when *w*(*x*, *y*, *z*_*T*_, *t*)<0. From this equation, *q*_*d*_ can be deduced.

Similarly, the *HDO* vapor tendency due to upward fluxes is calculated as

(20) $$M_{u}\cdot \left(R_{u}\cdot q_{u}-R_{1}\cdot q_{1}\right)\;=\;\frac{1}{n_{x}\cdot n_{y}\cdot n_{t}}\underset{x,y,t\in U}{\sum }\rho (z_{T})\cdot w(x,y,z_{T},t)\cdot \left(q(x,y,z_{T},t)\cdot R(x,y,z_{T},t)\;-\;\bar{q\cdot R}(z_{T})\right)$$

From this equation, *R*_*u*_ is deduced. In a similar way as for water vapor, *R*_*d*_ can also be deduced. Parameters *α*_*u*_ and *α*_*d*_ are deduced from Equations 11 and  12, respectively. They correspond to those that were calculated in Equations 2 and 3.

Parameter *F* is calculated as *F* = (*dq*/*dt*)_*mphy*_ where (*dq*/*dt*)_*mphy*_ is the water vapor tendency associated with phase changes: It is directly available in the outputs and is assumed to represent rain evaporation only in the SCL.

**Figure 9:**
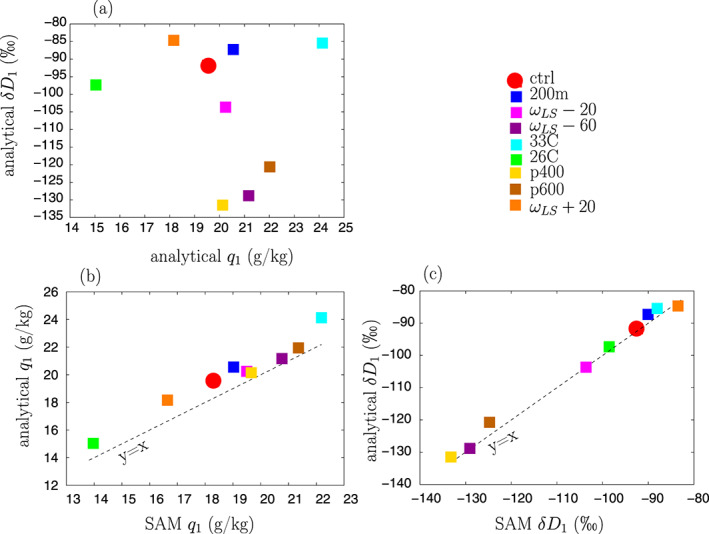
(a) *δD*_1_ as a function of *q*_1_ predicted by the analytical model for the different simulations. This can be directly compared to *δD*_1_ as a fuction of *q*_1_ simulated by SAM in Figure 3d. (b) *q*_1_ predicted by the analytical model as a function of *q*_1_ simulated by SAM. (c) Same as (b) but for *δD*.

The diagnosed parameters are summarized in Table 3. With these parameters, the analytical model is able to capture the simulated *q* and *δD*_*v*_ values and their sensitivity to SST and *ω*_*LSmax*_ (Figure 9). This gives us confidence to use it as an interpretative framework. In the next sections, we use the analytical model to better understand what controls *q*_1_ and *R*_1_.

**Table 3:** Parameter Values Diagnosed From the SAM Simulations and Used in the Analytical Model

Simulation name	*c*_*E*_ (kg/m^2^/day)	*M*_*u*_ (kg/m^2^/day)	*F* (mm/day)	*r*_*u*_ (%)	*r*_*d*_ (%)	*α*_*u*_ (‰)	*α*_*d*_ (‰)
ctrl	330	7,400	0.44	1.44	−0.38	71	105
*ω*_*LS*_ − 60	340	6,000	0.47	1.27	−0.45	251	326
*ω*_*LS*_ − 20	330	7,000	0.45	1.33	−0.41	117	182
*ω*_*LS*_ + 20	330	7,800	0.44	1.92	−0.28	42	63
26C	320	7,300	0.38	1.81	−0.18	74	133
33C	320	7,300	0.51	1.39	−0.28	70	84
*p*400	350	6,900	0.58	1.55	−0.38	218	315
*p*600	340	5,600	0.46	1.16	−0.38	254	314
200m	310	7,900	0.32	1.09	−0.19	73	83

*Note*. The value for SST corresponds to that prescribed in the simulation (Table 1). The values for *M*_*d*_ equal those for *M*_*u*_ within 2·10^−3^% in all simulations, so only *M*_*u*_ is indicated here. For *r*_*u*_ and *r*_*d*_, we give values of (*r*_*u*_ − 1)·100 and (*r*_*d*_ − 1)·100 to represent the deviations from *q*_1_ in %. For *α*_*u*_ and *α*_*d*_, we give values of (*α*_*u*_ − 1)·1,000 and (*α*_*d*_ − 1)·1,000 to express them in ‰.

### 3.4 What Processes Dry the SCL and Deplete Its Water Vapor Relative to Equilibrium With the Ocean?

**Figure 10:**
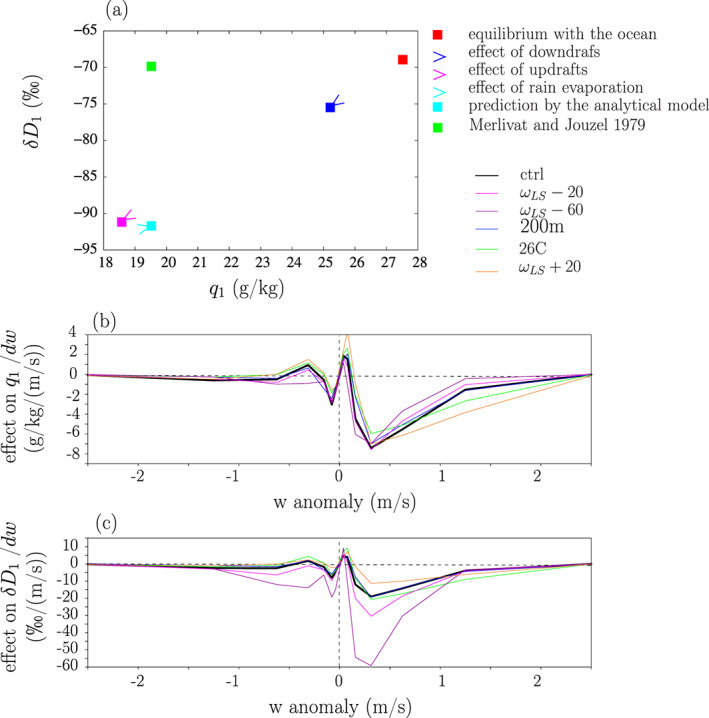
Decomposition of *q*_1_ and *δD*_1_ simulated by the analytical model for the ctrl simulation into the values that would be predicted if the air was in equilibrium with the ocean (red), the effect of downdrafts (blue), the effect of updrafts (magenta), and the effect of rain evaporation (cyan). (b) Decomposition of the effect of updrafts and downdrafts on *q*_1_ into the contributions from different bins of *w*, for the different simulations. Effect values are divided by the bin width for an easier visual integration: The area under the curve, calculated as $\int_{w=-\infty }^{+\infty }\frac{C(w)}{dw}\cdot dw$, where *C*(*w*) is the contribution from a given *w* bin, equals the total contribution for all updrafts and downdrafts (i.e., blue+pink arrows in (a)). (c) Same as (b) but for the effect on *δD*_1_. For (b) and (c), the effects are divided by the width of the *w* bins. For a clearer plot, Simulations *p*400 and *p*600 are omitted because they give results similar to *ω* − 60, and Simulation 33C is omitted because it gives results similar to ctrl.

What are the processes that make *q*_1_ and *δD*_1_ depart from equilibrium with the ocean in the ctrl simulation? At equilibrium, the air would be very moist and the water vapor *δD* as high as −69‰ (Figure 10, red). The contributions of updrafts, downdrafts and rain evaporation to the difference between *q*_1_ and the in equilibrium with the ocean are calculated as detailed in Text S4.

Updrafts and downdrafts respectively contribute to 83% and 29% of the drying from equilibrium with the ocean to *q*_1_ (Figure 10a, pink and green, Table 4). The drying by updrafts and downdrafts exceeds 100% because rain evaporation moistens by a small percentage. The stronger contribution of updrafts to the drying of the SCL is consistent with the stronger contribution of updrafts to the water export out of the SCL (section 2.6), which was caused by the presence of moderate downdrafts in moist regions of the domain. The relative contribution of updrafts and downdrafts to the drying of the SCL as a function of *w* (Figure 10b) echoes the water mass flux through the SCL top (Figure 5c). The main contribution comes from the moderate updrafts, which correspond to updrafts in the moist, shallow convective environment.

**Table 4:** Decomposition of the Difference Between *q*_1_ and *δD*_1_ Predicted by the Analytical Model and Those Predicted if in Equilibrium With the Ocean (Second Column, in g/kg for *q*_1_ and in ‰0 for *δD*_1_), Into the Contributions of Updrafts, Downdrafts, and Rain Evaporation, for the Different Simulations (Three Last Columns)

Simulation	Variable	Difference analytical model‐equilibrium with ocean	Effect of updrafts	Effect of downdrafts	Effect of rain evaporation
ctrl	*q*	−8.0 g/kg	− **6.6 (83%)**	−2.3 (29%)	0.9 (−12%)
	*δD*	−22.8 ‰	−15.7 **(69%)**	−6.5 (29%)	−0.5 (2%)
*ω*_*LS*_−60	*q*	−6.5 g/kg	−5.2 **(80%)**	−2.3 (36%)	1.1 (−16%)
	*δD*	−59.9 ‰	−38.6 **(64%)**	−18.6 (31%)	−2.8 (5%)
*ω*_*LS*_−20	*q*	−7.4 g/kg	−6.0 **(81%)**	−2.4 (32%)	1.0 (−14%)
	*δD*	−34.7 ‰	−22.1 **(64%)**	−11.3 (33%)	−1.4 (4%)
*ω*_*LS*_+20	*q*	−9.4 g/kg	−8.4 **(89%)**	−1.9 (20%)	0.9 (−9%)
	*δD*	−15.7 ‰	−12.7 **(81%)**	−3.1 (19%)	0.1 (0%)
26C	*q*	−6.8 g/kg	−6.4 **(94%)**	−1.2 (18%)	0.8 (−12%)
	*δD*	−24.7 ‰	−20.0 **(81%)**	−4.0 (16%)	−0.7 (3%)
33C	*q*	−8.5 g/kg	−7.5 **(88%)**	−2.1 (25%)	1.1 (−13%)
	*δD*	−19.1 ‰	−15.0 **(79%)**	−3.8 (20%)	−0.3 (2%)
*p*400	*q*	−7.8 g/kg	−6.7 **(86%)**	−2.3 (30%)	1.2 (−16%)
	*δD*	−62.8 ‰	−42.9 **(68%)**	−16.6 (26%)	−3.3 (5%)
*p*600	*q*	−5.6 g/kg	−4.7 **(83%)**	−2.0 (36%)	1.1 (−19%)
	*δD*	−52.0 ‰	−35.0 **(67%)**	−14.7 (28%)	−2.3 (4%)
200m	*q*	−6.7 g/kg	−5.9 **(89%)**	−1.5 (22%)	0.8 (−12%)
	*δD*	−18.3 ‰	−15.0 **(82%)**	−3.1 (17%)	−0.3 (1%)

*Note*. Contributions are given both in absolute values (g/kg for *q*_1_ and in ‰ for *δD*_1_) and in % of the total difference (within parentheses). Contributions larger than 50% are highlighted in bold. The sum of the three contributions in % should be between 99% and 101%, depending on rounding approximations.

Updrafts contribute to 69% of the depletion of the SCL from equilibrium to *δD*_1_. This contribution is closed to the contribution to the drying of the SCL, consistent with our expectation in section 3.2. Consistent with the tight relationship between *q* and *δD*, the relative contribution of updrafts and downdrafts to the depletion of the SCL as a function of *w* (Figure 10c) is almost the same as the relative contribution of updrafts and downdrafts to the drying of the SCL (Figure 10b). This is the first time that a major role of updrafts is highlighted for depleting the isotopic composition of the SCL water vapor.

Finally, rain evaporation adds a small amount of moisture back into the SCL, but its role is relatively minor. It does not significantly affect *δD*_*v*_, probably because the fraction of evaporated raindrops is intermediate between a small fraction (which would lead to a depleting effect) and a large fraction (which would lead to an enriching effect).

The green dot in Figure 10a corresponds to the (Merlivat & Jouzel, 1979) closure. This closure predicts a *δD*_1_ value that is very close to what we would expect from equilibrium with the ocean, because it neglects the isotopic impact of mixing with the overlying atmosphere.

These results are robust across all simulations. In all cases, updrafts make the strongest contribution to the difference between the analytical model predictions of *q*_1_ and *R*_1_ and those predicted by equilibrium with the ocean, and downdrafts make the second largest contribution (Table 4 and Figures 10b and 10c). But we can see that the large‐scale ascent impacts more strongly the effect of updrafts and downdrafts on *δD*_1_ than their effects on *q*_1_. This leads us to investigate in the next section the processes responsible for the impact of large‐scale ascent on *δD*_1_.

### 3.5 What Processes Are Responsible for the Amount Effect?

**Figure 11:**
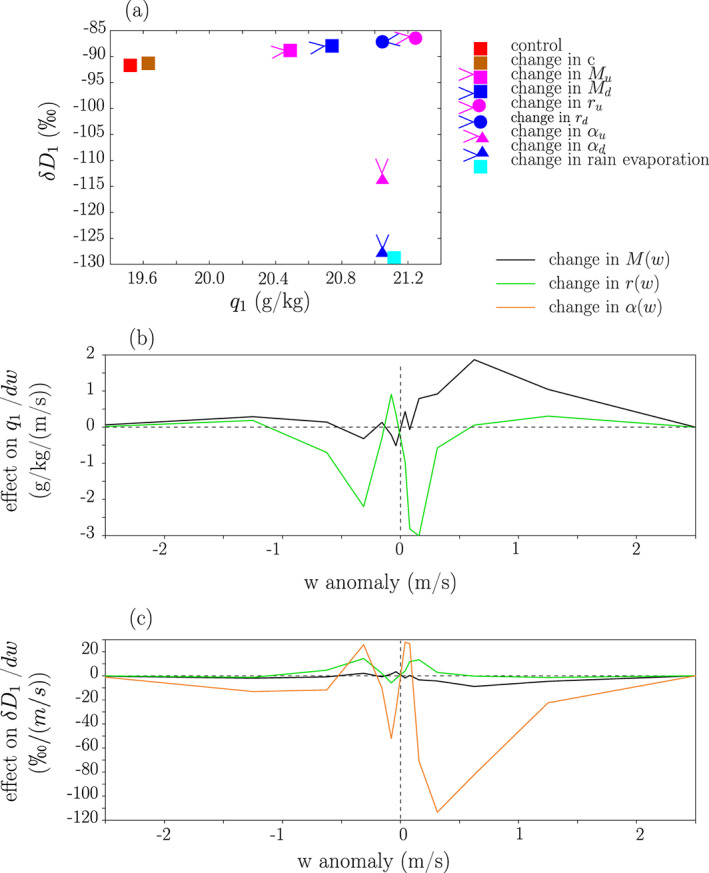
(a) Decomposition of the change in *q*_1_ and in *δD*_1_ from the ctrl (red square) to *ω* − 60 simulation (cyan circle), into the effect of the change of each parameter one by one. (b) Decomposition of the change in *q*_1_ from the ctrl to *ω* − 60 simulation into the effects of changes in *M* (black) and *r* parameters for different bins of vertical velocity *w*. Values are divided by the bin width for an easier visual integration: The area under the curve, calculated as $\int_{w=-\infty }^{+\infty }\frac{C(w)}{dw}\cdot dw$, where *C*(*w*) is the contribution from a given *w* bin, equals the total contribution for all updrafts and downdrafts. (c) Same as (b) but for the change in *δD*_1_. The effects of changes in *α* parameters is also added. For (b) and (c), the effects are divided by the width of the *w* bins.

What makes *q*_1_ more moist and *R*_1_ more depleted in the case of large‐scale ascent? To answer this question, we substitute each of the 9 parameters one by one in Equations 13 and 14 from their values in ctrl to their values in *ω*_*LS*_ − 60. Figure 11 compares ctrl to *ω*_*LS*_ − 60 and Table 5 compares different pairs of simulations.

**Table 5:** Decomposition of the Change in Variables *q* and *δD* From Simulation 1 to Simulation 2 (Column 4) Into the Effect of Changes of Individual Parameters in the Analytical Model (Next Columns)

Simulation 1	Simulation 2	Variable	Difference Simulation 1 − Simulation2	SST	*c*_*E*_	*M*_*u*_	*M*_*d*_	*r*_*u*_	*r*_*d*_	*α*_*u*_	*α*_*d*_	*F*
ctrl	*ω*_*LS*_−	*q*	1.60 g/kg	0	0.11	0.88	0.25	0.49	−0.20	0	0	0.08
	60				(6%)	**(55%)**	(15%)	(31%)	(−12%)			(4%)
		*δD*	—	0	0.4	2.3	1.0	1.3	−0.8	−28.7	−11.9	−0.8
			37.1 ‰		(−1%)	(−6%)	(−3%)	(−4%)	(2%)	**(77%)**	(32%)	(2%)
ctrl	*ω*_*LS*_−	*q*	0.67 g/kg	0	0.05	0.28	0.08	0.32	−0.09	0	0	0.03
	20				(8%)	(42%)	(11%)	(48%)	(−14%)			(5%)
		*δD*	—	0	0.2	0.7	0.3	0.9	−0.4	−8.8	−4.5	−0.4
			11.9 %0		(−2%)	(−6%)	(−3%)	(−7%)	(3%)	**(74%)**	(37%)	(3%)
ctrl	*ω*_*LS*_+	*q*	—	0	−0.01	−0.2	−0.05	−1.40	0.28	0	0	−0.00
	20		1.4 g/kg		(1%)	(15%)	(4%)	**(100%)**	(−20			(0%)
		*δD*	7.1 ‰	0	−0.0	−0.5	−0.2	−3.7	1.2	8.2	1.7	(6%)
					(0%)	(−8%)	(−3%)	(−52%)	(17%)	**(116%)**	(25%)	
ctrl	33C	*q*	4.61 g/kg	3.52	0.24	0.10	0.03	0.18	0.38	0	0	0.16
				**(76%)**	(5%)	(2%)	(1%)	(4%)	(8%)			(3%)
		*δD*	6.3 ‰	2.6	0.7	0.2	0.1	0.4	1.3	0.1	0.8	−0.1
				(41%)	(11%)	(4%)	(2%)	(6%)	(21%)	(2%)	(13%)	(−1%)
ctrl	26C	*q*	—	−3.92	−0.09	0.07	0.02	−0.88	0.45	0	0	−0.13
			4.48 g/kg	**(87%)**	(2%)	(−1%)	(0%)	(20%)	(−10%)			(3%)
		*δD*	−5.6 %0	−3.6	−0.4	0.2	0.1	−3.0	2.5	−0.7	−0.8	(−2%)
				**(64%)**	(8%)	(−4%)	(−2%)	**(53%)**	(−44%)	(13%)	(14%)	
*p*600	*p*400	*q*	—	0	0.08	−0.75	−0.24	−1.18	−0.00	0	0	0.26
			1.83 g/kg		(−4%)	(41%)	(13%)	**(64%)**	(0%)			(−14%)
		*δD*	—	0	1.0	−6.2	−2.5	−9.5	−0.0	6.9	−0.1	−0.5
			10.8 ‰		(−10%)	**(58%)**	(23%)	**(88%)**	(0%)	(−64%)	(0%)	(4%)

*Note*. Contributions are given both in absolute values (g/kg for *q*_1_ and in ‰ for *δD*_1_) and in % of the total difference (within parentheses). Contributions larger than 50% are highlighted in bold. The sum of the nine contributions in % should be between 99% and 101%, depending on rounding approximations.

The main process explaining the more moist SCL in *ω*_*LS*_ − 60 compared to ctrl is the smaller updraft flux *M*_*u*_ (55%), especially for moderate updrafts (Figure 11b). Generally, the total upward mass flux *M*_*u*_ increases when the surface precipitation decreases (Table 3 and Figure 7a). This result is consistent with previous LES studies showing that upward mass fluxes at cloud base do not necessarily increase with precipitation rate (Fletcher & Bretherton, 2010; Kuang & Bretherton, 2006). The smaller *r*_*u*_ in *ω* − 60, that is, smaller *q* anomalies in updrafts relative to the domain mean, also contributes to the moistening (31%, Table 5), especially for moderate updrafts (Figure 11b).

The main process explaining the more depleted SCL water vapor is the larger *α*_*u*_ (77%) and to a lesser extent the larger *α*_*d*_ (32%), that is, steeper *q* − *δD*_*v*_ relationships between updrafts, downdrafts and the domain mean. This effect is strongest for moderate updrafts (Figure 11c).

The exact contributions are slightly different when comparing ctrl to *ω*_*LS*_ − 20 or to *ω*_*LS*_ + 20, but what remains robust is the major effect of *α*_*u*_ to explain the more depleted vapor as large‐scale ascent increases or large‐scale descent decreases (Table 5). In summary, as large‐scale ascent increases, the *q* − *δD*_*v*_ relationship between updrafts and the domain mean is steeper, and thus updrafts deplete the SCL more efficiently.

### 3.6 What Processes Are Responsible for the Sensitivity to SST and to the Shape of Large‐Scale Ascent?

The same decomposition can be applied to any pair of simulations. For example, why is the 33C simulation more moist and more enriched than ctrl? The higher SST, through its direct effect on *q*_*sat*_(*SST*) and *α*_*eq*_(*SST*) is the main contributor to both the more moist SCL (74%) and more enriched water vapor. Conversely, the lower SST is the main contributor to the drier SCL (87%) and more depleted vapor (58%) in 26C compared to ctrl (Table 5).

Why is the *p*400 simulation drier and more depleted than the *p*600 simulation? This is mainly due to the larger *r*_*u*_, contributing to 64% of the *q*_1_ difference and 88% to the *δD*_1_ difference (Table 5). In other words,when the large‐scale vertical velocity is more top‐heavy, the humidity contrasts are stronger, and thus updrafts are more moist. Therefore, more moisture, which is enriched, is exported out of the SCL, leading to more efficient drying and depleting of the SCL.

We can notice that variations in SST, in draft fluxes (*M*_*u*_ or *M*_*d*_) or in humidity contrasts (*r*_*u*_ and *r*_*d*_) all lead to *q* and *δD* variations that are correlated. The only effect that can decorrelate *q*_1_ and *δD*_1_ are changes in the steepness of *q* − *δD*_*v*_ relationships between updrafts, downdrafts and the domain mean. (*α*_*u*_ and *α*_*d*_). This steepness strongly varies only when the large‐scale vertical velocity varies (section 3.5), explaining why the amount effect can be seen only when the large‐scale vertical velocity varies.

## 4 Conclusion

### 4.1 Isotopic Response to Dynamical and Thermodynamical Changes of Precipitation

Precipitation changes can be of dynamical (due to large‐scale circulation) or thermodynamical (due to changes in temperature) origin. In reality, the large‐scale circulation depends on the SST, with ascending motions favored over warmer SST (Bony et al., 2004; Sobel & Bretherton, 2000). Yet, decomposing precipitation changes into these two components can help to better understand precipitation changes in the future (Bony et al., 2013) or in the past (D'Agostino et al., 2019; Sun et al., 2016, 2018), and better understand the sources of intermodel spread in precipitation projections (Oueslati et al., 2016).

Our results show that the isotopic response to precipitation changes is different whether the changes in precipitation are of dynamical or thermodynamical origin. The amount effect can be observed only if the precipitation change is dynamical. If the precipitation change is thermodynamical, then the isotopic response is reversed and much smaller. These results are consistent with previous results with general circulation models (Risi, Bony, Vimeux, & Jouzel, 2010) and single column versions of GCMs (Bony et al., 2008). More generally, they may be consistent with the fact that several studies highlight the role of large‐scale circulation more than precipitation on the isotopic composition (Pausata et al., 2011). However, it remains to be checked in an isotopic GCM whether the decomposition of precipitation changes into their dynamic and thermodynamic component is a useful framework to better understand past isotopic signals.

### 4.2 Importance of Updrafts in Drying and Depleting the SCL

Our study highlights the key role of updrafts and downdrafts in determining the isotopic composition of the SCL. The relative depletion of downdrafts had already been cited as a reason for the amount effect (Kurita, 2013; Kurita et al., 2011; Risi et al., 2008; Risi, Bony, Vimeux, Chong, et al. 2010), but this is the first time that the relative enrichment of updrafts is highlighted as the key process by which convection depletes the SCL water vapor in heavy isotopes. The larger contribution of updrafts is due to the stronger correlation between velocity and humidity anomalies for updrafts than for downdrafts.

It would be interesting to document the properties of different kinds of updrafts and downdrafts in observations. This would require high‐frequency water vapor observations in updrafts and downdrafts areas of the SCL, colocated with vertical velocity measurements. Such observations were collected during the EUREC4A campaign (Bony et al., 2017) and will be very useful with this aim.

In GCMs, the properties of updrafts and downdrafts are calculated through parameterizations. Although the water vapor composition of downdrafts is usually explicitly calculated (Rio et al., 2019), few GCMs represent the fact the updrafts stem from areas of the SCL that are more moist and more enriched. To our knowledge, LMDZ5B is the only model that explicitly calculates cold pool properties (Grandpeix et al., 2010) and where deep convective updrafts stem from outside the cold pools (Hourdin et al., 2013). LMDZ6 is the only model in which both deep convective and shallow convective updrafts stem from outside cold pools (Hourdin et al., 2019). Therefore, it seems that an important component of the SCL moisture and isotopic budget is missing in most GCMs. It is surprising that GCMs are able to simulate the amount effect anyway. This may hint at some error compensations. For example, some GCMs show improved capacity to represent moist convection variability when they increase the effect of downdrafts (Del Genio et al., 2012; Mishra & Sahany, 2011), but this may be for the wrong reason (Thayer‐Calder & Randall, 2015; Torri & Kuang, 2016a). Excessively strong downdrafts may compensate for insufficient boundary layer turbulent mixing. The same kind of error compensation may happen for *δD* and the amount effect. In the future, it would be interesting to directly compare the properties of updrafts and downdrafts between an LES simulation and single column simulations with a GCM, by using conditional sampling techniques (Couvreux et al., 2010).

### 4.3 Perspectives: What Control the Steepness of *q*−*δD*_*v*_ Relationships Between Updrafts and Downdrafts?

Our study highlights the key role of the isotopic composition of downdrafts and updrafts relative to the domain mean. We showed that what is important to determine the efficiency of updrafts and downdrafts to deplete the SCL water vapor actually is the steepness of the *q* − *δD*_*v*_ relationship between updrafts, downdrafts and the domain mean. For example, if downdrafts are more depleted because they come from higher in altitude, they are also drier and thus they deplete the SCL water vapor less efficiently. Downdrafts deplete the SCL water vapor more efficiently only if they are more depleted because of a steeper *q* − *δD*_*v*_ relationship between downdrafts and the domain mean.

We thus identified the key role of the steepness of the *q* − *δD*_*v*_ relationship between updrafts, downdrafts and the domain mean. This is a big step forward, but actually it only pushes the problem a bit further. What controls this steepness? We showed that it was related to the steepness of the domain mean *q* − *δD*_*v*_ relationship in the vertical. Now, what controls *δD*_*v*_ vertical profiles? A comprehensive understanding of what controls the SCL water vapor thus requires to better understand what controls isotopic vertical gradients, a conclusion that was already reached in (Risi et al., 2019). In observations, *δD* vertical profiles can be very diverse (Salmon et al., 2019; Sodemann et al., 2017). Based on observations, simple models or models with parameterized convection, previous studies have suggested possible roles for rain evaporation (Worden et al., 2007), vertical mixing (Galewsky & Hurley, 2010; Risi et al., 2012), convective detrainment (Bony et al., 2008; Kuang et al., 2003; Schmidt et al., 2005), but the relative importance of these processes has never been quantified. Therefore, our next study will aim at quantifying the relative importance of these processes using LES simulations.

## Supporting information



Supporting Information S1

Movie S1

Movie S2

## Data Availability

Information on SAM can be found online (on this web page: http://rossby.msrc.sunysb.edu/marat/ SAM.html). All simulation outputs used in this article are available from the PANGEA data repository (https://doi.org/10.1594/PANGAEA.918620).
