# Supplementary material for: What Controls the Water Vapor Isotopic Composition Near the Surface of Tropical Oceans? Results From an Analytical Model Constrained by Large‐Eddy Simulations
Source: J Adv Model Earth Syst. 2020 Aug 12;12(8):e2020MS002106. doi: 10.1029/2020MS002106 (PMC7507762; doi:10.1029/2020MS002106)
Supplement: Supplementary file 1 — Supporting Information S1 [file JAME-12-e2020MS002106-s001.pdf]

Supporting Information for "What controls the water vapor isotopic composition near the surface of tropical oceans? Results from an analytical model constrained by large-eddy simulations"

Camille Risi [1], Caroline Muller [1], Peter Blossey [2]

[1]Laboratoire de Meteorologie Dynamique, IPSL, CNRS, Ecole Normale Superieure, Sorbonne Universite, PSL Research University, Paris, France

[2]Department of Atmospheric Sciences, University of Washington, Seattle, USA

Contents of this file

1. Text S1: Relative contributions of the updrafts to the water export out of the sub-cloud layer

2. Text S2: Equations for rain evaporation

3. Text S3: Extension of the analytical model to consider any number of draft categories

4. Text S4: Calculating the contributions to the drying and to the depletion of the SCL relative to equilibrium with the ocean

Additional Supporting Information (Files uploaded separately)

1. Movie S1: Video of the control simulation

2. Movie S2: Video of the $\omega_{LS} - 60$ simulation

Introduction

This supporting information gives details about some equations that are useful to interpret the water budget of the sub-cloud layer (Text S1), to derive the analytical model for this budget (Text S2 and S3) or to estimate the contributions from different processes to the sub-cloud layer properties (Text S4).

We also give videos that gather snapshots of two of our LES simulations: ctrl and $\omega_{LS}-60$ (Movies S1 and S2). These videos are created by gathering the 132 last output time steps of these simulations, with an output frequency of 15 minutes. The videos show the maps of vertical velocity anomalies at the SCL top (a), precipitation rate at the surface (b), specific humidity anomalies (c) and water vapor δD anomalies (d) at the the lowest model level. Anomalies are relatively to the average over the domain and over the 132 time steps.

Text S1: Relative contributions of the updrafts to the water export out of the sub-cloud layer

Both updrafts and downdrafts contribute to exporting water out of the sub-cloud layer (SCL). The mass conservation imposes that the air leaving the SCL through updrafts has to come back down into the SCL through downdrafts. Does this mean that the updrafts and downdrafts contribute equally to the export of water out of the SCL, i.e. 50%-50%? The goal of this section is to express the relative contributions of the updrafts to the water export out of the SCL, and to understand why it could deviate from 50%.

At each location and time step, the flux f of water out of the SCL, expressed in $kg/m^2/s$ is calculated as:

$$f(x,y,t) = \rho(z_T) \cdot w(x,y,z_T,t) \cdot (q(x,y,z_T,t) - \overline{q}(z_T))$$

where $\rho(z)$ is the volumetric mass assumed to be an unique function of altitude, $w(x,y,z_T,t)$ and $q(x,y,z_T,t)$ are the grid-scale vertical velocity and specific humidity at SCL top, and $\overline{q}(z_T)$ is the domain-mean, time-mean specific humidity at SCL top.

The time-mean, domain-mean flux $\overline{f}$ of water out of the SCL is:

$$\overline{f} = \frac{1}{n}\sum_{x,y,t} \rho(z_T)\cdot w(x,y,z_T,t)\cdot (q(x,y,z_T,t) - \overline{q}(z_T))$$

where $n = n_x \cdot n_y \cdot n_t$, n_x and n_y are the numbers of points in the x and y dimensions, n_t is the number of snapshots taken into account (10, with one snapshot every day). The time-mean, domain-mean flux contributed by updrafts is:

$$\overline{f_u} = \frac{1}{n}\sum_{x,y,t\in U} \rho(z_T)\cdot w(x,y,z_T,t)\cdot (q(x,y,z_T,t) - \overline{q}(z_T))$$

where U is the ensemble of (x,y,t) where and when $w(x,y,z_T,t) > 0$. Similarly, the time-mean, domain-mean flux contributed by downdrafts only is:

$$\overline{f_d} = \frac{1}{n}\sum_{x,y,t\in D} \rho(z_T)\cdot w(x,y,z_T,t)\cdot (q(x,y,z_T,t) - \overline{q}(z_T))$$

where D is the ensemble of (x,y,t) where and when $w(x,y,z_T,t) \leq 0$. We thus have $\overline{f} = \overline{f_u} + \overline{f_d}$. The goal of this section is to express the relative contributions of the updrafts to the water export out of the SCL, i.e. $\overline{f_u}/\overline{f}$.

Let's first calculate $\overline{f_u}$. We make a Reynolds decomposition of q and w in updrafts:

$$q(x,y,z_T,t) = \overline{q_u} + q'(x,y,z_T,t)$$

and

$$w(x,y,z_T,t) = \overline{w_u} + w'(x,y,z_T,t)$$

where the $\overline{q_u}$ and $\overline{w_u}$ are averages over U:

$$\overline{q_u} = \frac{\sum_{x,y,t \in U} q(x,y,z_T,t)}{\sum_{x,y,t \in U} 1} \tag{1}$$

$$\overline{w_u} = \frac{\sum_{x,y,t \in U} w(x,y,z_T,t)}{\sum_{x,y,t \in U} 1} \tag{2}$$

and $q'(x,y,z_T,t)$ and $w'(x,y,z_T,t)$ are anomalies of q and w relative to the averages over U. By definition of anomalies, we have $\sum_{x,y,t \in U} q'(x,y,z_T,t) = 0$ and $\sum_{x,y,t \in U} w'(x,y,z_T,t) = 0$.

We get:

$$\overline{f_u} = \rho(z_T) \cdot a_u \cdot \overline{w_u} \cdot (\overline{q_u} - \overline{q}) + \frac{\rho(z_T)}{n} \cdot \sum_{x,y,t \in U} w'(x,y,z_T,t) q'(x,y,z_T,t)$$

where $a_u = \frac{1}{n} \sum_{x,y,t \in U} 1$ is the fractional area covered by updrafts. The second term in the right-hand side represents the co-variance between w' and q' over U . If we re-write it as a function of the correlation coefficient, we get:

$$\overline{f_u} = \rho(z_T) \cdot a_u \cdot \overline{w_u} \cdot (\overline{q_u} - \overline{q}) + \rho(z_T) \cdot a_u \cdot r_U(w', q') \cdot \sigma_U(w') \cdot \sigma_U(q') \tag{3}$$

where $r_U(w', q')$ is the correlation coefficient between w' and u' over U, $\sigma_U(w')$ is the standard deviation of w' and $\sigma_U(q')$ is the standard deviation of q' over U.

Similarly, we have:

$$\overline{f_d} = \rho(z_T) \cdot a_d \cdot \overline{w_d} \cdot (\overline{q_d} - \overline{q}) + \rho(z_T) \cdot a_d \cdot r_D(w', q') \cdot \sigma_D(w') \cdot \sigma_D(q') \tag{4}$$

Domain-mean, time-mean quantities can be expressed as a function of quantities averaged over updrafts and downdrafts:

$$a_u + a_d = 1$$

$$a_u \cdot \overline{q_u} + (1 - a_u) \cdot \overline{q_d} = \overline{q}$$

$$a_u \cdot \overline{w_u} + (1 - a_u) \cdot \overline{w_d} = \overline{w} = 0$$

Using these equations to express a_d, $\overline{q_d}$ and $\overline{w_d}$ as a function of a_u, $\overline{q_u}$ and $\overline{w_u}$ respectively, and injecting these values into equation 4, we get:

$$\overline{f_u}/\overline{f} = \frac{(1 - a_u) \cdot \overline{w_u} \cdot (\overline{q_u} - \overline{q}) + (1 - a_u) \cdot r_U(w', q') \cdot \sigma_U(w') \cdot \sigma_U(q')}{\overline{w_u} \cdot (\overline{q_u} - \overline{q}) + (1 - a_u) \cdot r_U(w', q') \cdot \sigma_U(w') \cdot \sigma_U(q') + (1 - a_u)^2 \cdot r_D(w', q') \cdot \sigma_D(w') \cdot \sigma_D(q')/a_u} \tag{5}$$

Therefore, the relative contributions of the updrafts to the water export out of the SCL, $\overline{f_u}/\overline{f}$, depends on the fraction of the domain covered by updrafts a_u. If updrafts cover a small fraction of the domain ($a_u \ll a_d$), their velocity and humidity anomalies relative to the mean are larger than those in downdrafts ($|\overline{w_u} \gg |\overline{w_d}|$ and $|\overline{q_u} - \overline{q}| \gg |\overline{q_d} - \overline{q}|$). The effects of the large velocity and humidity anomalies overwhelm the effect of the small fraction of the domain, so that $\overline{f_u}/\overline{f}$ scales with $1 - a_u$.

The relative contribution of the updrafts to the water export out of the SCL also depends on the joint probability density function of w' and q' for updrafts and downdrafts. In particular, it depends crucially on the correlations between w' and q' for updrafts and downdrafts.

If the correlations between between w' and q' were null, i.e. $r_U(w', q') = r_D(w', q') = 0$, then we would simply have $\overline{f_u}/\overline{f} = (1 - a_u)$. Since the updrafts cover nearly half of the

domain in our simulations, we would thus have $\overline{f_u}/\overline{f} \simeq 0.5$, i.e. updrafts and downdrafts would contribute equally to the water export out of the SCL.

In reality, correlations between between w' and q' are positive. The relative contribution of the updrafts to the water export increases as $r_U(w', q')$ increases and as $r_D(w', q')$ decreases. Since w' and q' are better correlated for updrafts than for downdrafts in our simulations, this explains why $\overline{f_u}/\overline{f}$ is higher than 50%.

Text S2: Equations for rain evaporation

The goal of this SI is to detail the equations allowing us to calculate the isotopic ratio of the rain evaporation flux, R_F.

We assume that rain drops evaporate like one drop in a homogeneous environment, following Stewart (1975)'s equation. We assume that the environment has a constant relative humidity $h_{ev} = \frac{q_1}{q_{sat}(T_1)}$ and a constant water vapor isotopic ratio R_1. The fractionation during rain evaporation depends on the equilibrium fractionation factor $\alpha_{eq}(T(z_T))$, reflecting the distribution of isotopes between liquid and vapor at equilibrium, and on the kinetic fractionation factor α_{Kev}, which reflects the molecular diffusivities of isotopes.

In SAM, the formulation for the kinetic fractionation is sophisticated to account for the diffusive conditions of the vapor near the surface of rain drops. Here we simply use Stewart (1975)'s formulation:

$$\alpha_{Kev} = \left(\frac{D_{HDO}}{D_{H_2O}} \right)^{0.58}$$

where D_{HDO} and D_{H_2O} are the molecular diffusivities of HDO and H_2O respectively.

Stewart (1975)'s equation estimates the isotopic composition of the rain after evaporation, R_p, as:

$$R_p = R_{pT} \cdot (1 - f_{ev})^{\beta} + \gamma \cdot R_1 \left(1 - (1 - f_{ev})^{\beta}\right)$$

with

$$\beta = \frac{1 - \alpha_{eq}(T(z_T)) \cdot \alpha_{Kev} \cdot (1 - h_{ev})}{\alpha_{eq}(T(z_T)) \cdot \alpha_{Kev} \cdot (1 - h_{ev})}$$

and

$$\gamma = \frac{\alpha_{eq}(T(z_T)) \cdot h_{ev}}{1 - \alpha_{eq}(T(z_T)) \cdot \alpha_{Kev} \cdot (1 - h_{ev})}$$

We define R_{pT} as the isotopic ratio of the rain at the SCL top, before being affected by rain evaporatio in the SCL.

The isotopic composition of the rain evaporation flux R_F can be calculated by a mass balance:

$$R_F = \frac{R_{pT} - (1 - f_{ev}) \cdot R_p}{f_{ev}}$$

We thus get:

$$R_F = \frac{R_{pT}\left(1 - (1 - f_{ev})^{\beta+1}\right) - (1 - f_{ev}) \cdot \left(1 - (1 - f_{ev})^{\beta}\right) \cdot \gamma \cdot R_1}{f_{ev}} \tag{6}$$

To estimate R_{pT}, we assume that the isotopic composition of the rain at the SCL top is in equilibrium with the domain-mean water vapor. This is justified by the fact that in SAM simulations, the isotopic composition of the rain is close to equilibrium with the SCL water vapor. In reality, the rain and the vapor may not always be in equilibrium with the vapor because (1) the rain falls in downdrafts that are expected to be more depleted than R_1, and (2) the rain falls from condensate formed in altitude where the vapor is more depleted, and may not have the time to fully equilibrate (Lee & Fung, 2008), and (3) the

rain may be isotopically enriched by rain evaporation (Tremoy et al., 2014). Here, for the sake of simplicity, the isotopic composition of the rain at the SCL top is simply assumed to be in equilibrium with the SCL water vapor:

$$R_{pT} = \alpha_{eq}(T(z_T)) \cdot R_1 \tag{7}$$

We look for a solution of the form:

$$R_F = A \cdot R_1 \tag{8}$$

Combining equations 6, 7 and 8, we get:

$$A = \frac{\alpha_{eq}(T(z_T)) \cdot \left(1 - (1 - f_{ev})^{\beta+1}\right) - \gamma \cdot (1 - f_{ev}) \cdot \left(1 - (1 - f_{ev})^{\beta}\right)}{f_{ev}}$$

Text S3: Extension of the analytical model to consider any number of draft categories

We recall the equations of the analytical model to predict q_1 and R_1:

$$q_1 = \frac{q_{sat}^{surf}(SST) + F/c}{1 + (1/c) \cdot (M_u \cdot (r_u - 1) - M_d \cdot (r_d - 1))} \tag{9}$$

$$R_1 = \frac{R_{oce}/\alpha_{eq}(SST)}{h_1 + \alpha_K \cdot (1 - h_1) \cdot \left((1 + F/E) \cdot \frac{M_u(r_u^{\alpha_u} - 1) - M_d \cdot \left(r_d^{\alpha_d} - 1\right)}{M_u(r_u - 1) - M_d \cdot (r_d - 1)} - F \cdot A/E\right)} \tag{10}$$

Equations (9) and (10) can be extended to consider any number n of draft categories:

$$q_1 = \frac{q_{sat}^{surf}(SST) + F/c}{1 + (1/c) \cdot (\sum_{i=1}^{n} M_i \cdot (r_i - 1))} \tag{11}$$

$$R_1 = \frac{R_{oce}/\alpha_{eq}(SST)}{h_1 + \alpha_K \cdot (1 - h_1) \cdot \left((1 + F/E) \cdot \frac{\sum_{i=1}^{n} M_i \cdot (r_i - 1)}{\sum_{i=1}^{n} M_i \cdot \left(r_i^{\alpha_i} - 1\right)} - F \cdot A/E \right)} \quad (12)$$

where M_i, r_i and α_i are the mass fluxes, humidity ratios and effective fractionations for each category i. M_i are positive for upward fluxes and negative for downward fluxes.

All these parameters can be diagnosed from SAM simulations as explained in section 3.2 of the main text, except that the sums are calculated only for $x, y, t \in I$, where I is the ensemble of (x, y, t) belonging to category i.

Text S4: Calculating the contributions to the drying and to the depletion of the SCL relative to equilibrium with the ocean

What processes dry the SCL relative to equilibrium with the ocean? To answer this question raised in section 3.4 of the main text, we quantify the contributions of the updrafts, downdrafts and rain evaporation to the difference between the SCL specific humidity q_1 and the specific humidity in equilibrium with the ocean q_{eq}. According to our analytical model (equation 9), q_1 is a function of several variables including F, M_u and M_d. Let's note $q_f(M_u, M_d, F)$ this function. The specific humidity when accounting for all effects is $q_1 = q_f(M_u, M_d, F)$ and that in equilibrium with the ocean is $q_{eq} = q_f(0, 0, 0)$.

The relative contributions of updrafts, downdrafts and rain evaporation (c_{up1}, c_{down1}, c_{ev}) can be calculated by adding the effects of updrafts, downdrafts and rain evaporation one by one:

$$c_{up1} = q_f(M_u, 0, 0) - q_f(0, 0, 0)$$

$$c_{down1} = q_f(M_u, M_d, 0) - q_f(M_u, 0, 0)$$

$$c_{ev} = q_f(M_u, M_d, F) - q_f(M_u, M_d, 0)$$

Alternatively, the relative contributions of updrafts and downdrafts (c_{up2}, c_{down2}) can be calculated by adding the effects of downdrafts first, then updrafts:

$$c_{down2} = q_f(0, M_d, 0) - q_f(0, 0, 0)$$

$$c_{up2} = q_f(M_u, M_d, 0) - q_f(0, M_d, 0)$$

Due to non-linear effects, $c_{up1} \neq c_{up2}$ and $c_{down1} \neq c_{down2}$. To avoid the sensitivity to the arbitrary choice of which effect we add first, we estimate the contributions of updrafts and downdrafts as follows:

$$c_{up} = \frac{c_{up1} + d_{up2}}{2}$$

$$c_{down} = \frac{c_{down1} + d_{down2}}{2}$$

We check that the sum of all contributions accounts for 100 % of the $q_1 - q_{eq}$ difference:

$$c_{up} + d_{down} + c_{ev} = q_1 - q_{eq}$$

The relative contributions of the updrafts, downdrafts and rain evaporation to the depletion of the SCL water vapor are quantified the same way, by replacing q by the isotopic ratio R.

Movie S1: Video of the control simulation

Movie S2: Video of the $\omega_{LS} - 60$ simulation

References

Lee, J.-E., & Fung, I. (2008). "Amount effect" of water isotopes and quantitative analysis of post-condensation processes. *Hydrological Processes*, *22 (1)*, 1-8.

Stewart, M. K. (1975, March). Stable isotope fractionation due to evaporation and isotopic exchange of falling waterdrops: Applications to atmospheric processes and evaporation of lakes. *J. Geophys. Res.*, *80*, 1133-1146.

Tremoy, G., Vimeux, F., Soumana, S., Souley, I., Risi, C., Cattani, O., ... Oi, M. (2014). Clustering mesoscale convective systems with laser-based water vapor delta18O monitoring in Niamey (Niger). *J. Geophys. Res.*, *119(9)*, 5079-5103, DOI: 10.1002/2013JD020968.
